# Machine Learning-Assisted Analysis of Chloride-Enhanced BiOCl/DSA Anode for Synergistic Denitrification and Tetracycline Degradation: Performance, Mechanisms, and Product Regulation

**DOI:** 10.3390/nano16191219

**Published:** 2026-09-27

**Authors:** Yuankun Liu, Gangyi Sun, Zonglin Li, Heyang Wang, Yufeng He

**Affiliations:** Municipal Engineering Department, College of Civil Engineering and Architecture, Beijing University of Technology, Beijing 100124, China

**Keywords:** BiOCl/DSA anode, denitrification, fuzzy neural network (FNN), tetracycline (TC), wastewater treatment

## Abstract

Electrochemical co-treatment of nitrogen-containing and antibiotic wastewater is constrained by competitive oxygen evolution and insufficient chlorine evolution selectivity, while the resulting complex reaction network remains difficult to resolve. Therefore, a chloride-enhanced BiOCl/DSA anode coupled with a ZVI/Co_3_O_4_/GF cathode was constructed for deep denitrification and simultaneous tetracycline (TC) degradation. A fuzzy neural network (FNN) was further used to predict NH_4_^+^-N formation and conversion. The effects of initial NO_3_^−^-N and TC concentrations, KCl concentration, pH and cathode potential were systematically examined. At each tested KCl concentration, the BiOCl/DSA anode yielded a higher free residual chlorine concentration than the unmodified DSA anode. Relative to the commercial DSA anode, the BiOCl/DSA anode increased apparent dissolved inorganic nitrogen (DIN) removal from 60.17% to 71.89%, decreased NH_4_^+^-N accumulation from 44.54 to 28.48 mg/L, and increased TC removal from 74.51% to 100%. Complete DIN removal was achieved at 1000 mg/L KCl and a cathode potential of −1.4 V versus Ag/AgCl, while TC was efficiently degraded within 80 min. The BiOCl layer may promote chloride adsorption and chlorine-radical generation, thereby facilitating NH_4_^+^ oxidation and TC degradation. Among the four models compared, FNN showed the best internal fitting performance under the random 80:20 data split (test-set R^2^ = 0.9507). Given the limited size of the dataset, the model was used only for exploratory analysis within the range covered by the experimental data, and its generalizability to new operating conditions remains to be established using independent validation data.

## 1. Introduction

The co-occurrence of nitrate and antibiotic contaminants in wastewater, particularly from agricultural and pharmaceutical activities, represents a major environmental challenge because of their persistence and ecological risks [1,2]. Electrochemical technologies have emerged as promising approaches for pollutant removal because of their operational flexibility and environmental compatibility [3,4,5,6]. However, conventional electrochemical nitrate reduction commonly produces NH_4_^+^ as the predominant terminal product rather than selectively producing N_2_. Because the subsequent conversion of NH_4_^+^ is strongly influenced by reaction conditions and the electrochemical environment, precise regulation of nitrogen-conversion pathways remains a key challenge in electrochemical denitrification [7,8,9].

To overcome this limitation, electrochlorination has been proposed [10,11]. This process generates reactive chlorine species in situ, including Cl_2_, HOCl, and Cl•, which exhibit strong oxidation capacities toward ammonium and organic contaminants. Its efficiency, however, depends largely on the anodic chlorine evolution reaction (ClER), which is often impeded by the competing oxygen evolution reaction (OER), particularly at low chloride concentrations.

Dimensionally stable anodes (DSAs), typically coated with IrO_2_–Ta_2_O_5_, are widely used to drive chlorine evolution in practical electrochemical systems because of their excellent stability [12]. Nevertheless, conventional DSAs retain limited ClER selectivity at low Cl^−^ concentrations, and substantial electrical energy is consumed by the unproductive OER side reaction. Advanced anodes with enhanced chloride activation and ClER selectivity are therefore needed. Bismuth oxychloride (BiOCl) has a distinctive layered structure and an intrinsic internal electric field, which confer strong halide affinity and favourable interfacial charge-transfer properties. Its tunable defect structure, efficient charge-carrier separation and abundant surface-active sites have attracted considerable attention. Chauhan et al. [13] reviewed defect-engineering strategies for BiOCl and their photocatalytic applications, showing that surface defects and an internal electric field can promote interfacial charge transfer and reactant activation. Xu et al. [14] further showed that BiOCl can use chloride in the reaction system to generate reactive chlorine species for efficient ammonium removal. These properties suggest that BiOCl modification may promote chloride adsorption and activation, improve ClER performance and regulate nitrogen-conversion pathways.

However, the role of BiOCl/DSAs in electrochemical ClER and the regulation exerted by individual operating factors during nitrate reduction remain insufficiently understood. As a key intermediate, NH_4_^+^-N accumulation is governed not only by NO_3_^−^-N reduction but also by cathodic production, anodic consumption by reactive chlorine species, chloride migration, voltage, and pH. Consequently, NH_4_^+^-N concentration displays marked nonlinearity and uncertainty. Conventional response surface methodology and multivariate regression rely on predefined functional forms and are suitable mainly for identifying dominant factors and low-order interactions. They are inadequate for representing the strongly nonlinear, non-monotonic responses and gradual boundaries arising from cathodic nitrate reduction, anodic reactive-chlorine oxidation, ClER/OER competition, and pH regulation.

Data-driven machine learning (ML) has shown considerable potential for resolving multivariable coupling and nonlinear responses in advanced water-treatment processes [15,16]. Zhang et al. [17] showed that back-propagation (BP) neural networks and optimized BP models can capture nonlinear relationships between operational variables, including current density, pH, electrode material and pollutant concentration, and electrochemical treatment performance. Conventional BP models, however, rely on randomly initialized weights and thresholds and can become trapped in local optima. Although GA-BP and PSO-BP improve convergence by optimizing initial parameters, they remain deterministic mappings. In electrochemical systems, NH_4_^+^-N concentration is jointly affected by NO_3_^−^-N concentration, cathodic reduction, anodic oxidation by reactive chlorine species, chloride migration, cathode potential and pH. A suitable model must therefore describe nonlinear mappings while identifying gradual boundaries and uncertain responses arising from competing processes. Fuzzy neural networks (FNNs) combine rule-based fuzzy logic with the self-learning capacity of neural networks and can identify gradual responses and handle uncertainty under multivariable coupling. Qiao et al. and Han et al. [18,19] reported that FNNs accurately identify complex dynamic changes and manage system uncertainty, providing modelling performance superior to conventional neural networks in multivariable systems. FNNs are thus appropriate for predicting NH_4_^+^-N concentration and describing the relationship between NH_4_^+^-N formation and oxidative consumption under the coupled effects of chloride concentration, cathode potential, pH and initial NO_3_^−^-N concentration.

Building on our previous work with a ZVI/Co_3_O_4_/GF composite cathode exhibiting favourable nitrate-reduction activity [20], this study constructed a BiOCl-modified DSA anode (BiOCl/DSA) and a chloride-enhanced electrochemical system for simultaneous nitrate reduction and tetracycline degradation. The effects of BiOCl modification on anodic polarization, free residual chlorine concentrations, and nitrogen-species transformation were evaluated. BP, GA-BP, PSO-BP, and FNN models were compared using reaction time, KCl concentration, initial NO_3_^−^-N concentration, cathode-potential magnitude, and pH as inputs and NH_4_^+^-N concentration as the output. The models were used to compare their internal fitting performance and to explore NH_4_^+^-N response trends within the range covered by the experimental data. This work evaluates the performance of the BiOCl/DSA system for chloride-assisted electrochemical denitrification and TC removal.

## 2. Materials and Methods

### 2.1. Materials

Potassium nitrate (KNO_3_), cobalt nitrate hexahydrate [Co(NO_3_)_2_·6H_2_O], sodium dodecyl sulfate (SDS), boric acid (H_3_BO_3_), sodium sulfate (Na_2_SO_4_), ammonium sulfate [(NH_4_)_2_SO_4_], ferrous sulfate (FeSO_4_), ascorbic acid, glucose pentahydrate, ethanol, potassium chloride (KCl), ethylenediaminetetraacetic acid, potassium bromate, tert-butanol, N,N-diethyl-p-phenylenediamine, Nessler reagent, naphthylethylenediamine dihydrochloride, phosphoric acid, sulfanilamide, hydrochloric acid, sulfamic acid, potassium sodium tartrate and tetracycline (TC) were purchased from Beijing Jiashiteng Trading Co., Ltd. (Beijing, China). All chemicals were of analytical grade and used without further purification. Graphite felt (GF) was purchased from Tianjin Carbon Factory(Tianjin, China).

### 2.2. Electrode Preparation

The BiOCl/DSA anode was prepared by a wet-chemical synthesis method [21]. BiOCl was deposited onto the surface of a commercial Ir–Ta mixed-metal-oxide DSA anode purchased from Kunshan Yiwanlin Environmental Protection Materials Co., Ltd. (Kunshan, China) to modify the anodic response and promote ammonium removal. The commercial DSA anode was electrodeposited for 5 min at 5 mA cm^−2^ in an ethylene glycol solution containing 10 mM Bi(NO_3_)_3_·5H_2_O (ethylene glycol:water = 2:1), rinsed with deionized water and dried at 60 °C. The electrode was immersed in 1 M NaCl at pH 2 for 24 h, rinsed with ultrapure water and dried at 60 °C. It was then calcined in a tube furnace at 500 °C for 2 h and cooled to room temperature. Finally, the electrode was fixed by electrodeposition for 3 h at 10 mA cm^−2^ in a chloride-containing solution, rinsed and retained for subsequent use. The same BiOCl/DSAs were repeatedly used throughout the experimental campaign, rather than using a newly prepared anode for each batch experiment. The preparation procedure is shown in Figure S1. The ZVI/Co_3_O_4_/GF composite cathode was prepared as described in Section S1.

### 2.3. Analytical Methods

Details of electrode characterization, electrochemical measurements and electrochemical experiments are provided in Sections S2–S4.

### 2.4. Construction and Validation of Machine-Learning Models

To compare the internal fitting performance of different models for NH_4_^+^-N concentration, FNN, BP, GA-BP, and PSO-BP models were constructed and compared; their detailed architectures are provided in Sections S5–S8. The dataset comprised 90 observations from periodic sampling experiments in a single-chamber reactor (Table S1). These observations included multiple sampling times from several experimental runs and eligible data from the experiments reported in the main text and additional experiments conducted using the same reactor, electrode system, and analytical methods. Reaction time, KCl concentration, initial NO_3_^−^-N concentration, cathode-potential magnitude (|Ecathode|, V versus Ag/AgCl) and pH were used as inputs, and NH_4_^+^-N concentration was the output. The positive values of 1.2–1.8 V in the dataset represent the absolute magnitudes of cathode potentials ranging from −1.2 to −1.8 V versus Ag/AgCl and do not represent the cell voltage. The dataset was divided into training and test sets at an 80:20 ratio, and model training and testing were repeated using 30 random data splits.

All four models used the same dataset and input–output variables. BP was used as a reference feed-forward neural network. GA-BP and PSO-BP optimized the initial BP weights and thresholds using a genetic algorithm and particle-swarm optimization, respectively. FNN combined fuzzy logic with a neural network to fit the nonlinear associations between operating parameters and NH_4_^+^-N concentration within the observed dataset; its structure is shown in Figure S2. The FNN used 10 fuzzy rules and comprised five input-layer nodes, 50 membership-function-layer nodes, 10 fuzzy-rule-layer nodes, 10 defuzzification-layer nodes and one output-layer node. Parameters were iteratively updated using gradient descent.

Model performance was evaluated using MAE, MSE, RMSE and R^2^. Lower MAE, MSE and RMSE values and an R^2^ value closer to unity indicate better prediction performance. The equations for these metrics are provided in Section S9, and the model with the best overall performance was selected for subsequent analysis.

## 3. Results and Discussion

### 3.1. Physicochemical Characteristics of the BiOCl/DSA Electrode

#### 3.1.1. SEM Analysis of BiOCl/DSA Morphology

Figure 1a presents an SEM image of the DSA anode substrate (MMO). Distinct flake-like structures and microcracks were observed, which are commonly associated with thermal-stress release in metal-oxide coatings during thermal treatment. After BiOCl loading (Figure 1b), abundant flake-like or layered structures were uniformly distributed across the MMO surface. The lateral dimensions of these layered structures were approximately 1–3 μm, similar to the morphology previously reported for BiOCl-functionalized DSAs prepared using the same procedure [21,22]. These observations confirm the formation of a uniform layered coating with good surface coverage, although SEM alone cannot determine its precise phase composition after calcination. The layered morphology may increase the available interfacial area and facilitate reactant mass transfer at the electrode surface, thereby contributing to the observed electrochemical response and pollutant-removal performance.

#### 3.1.2. Electrochemical Performance of BiOCl/DSA

Figure 2 shows the cyclic voltammetry curves of BiOCl/DSA (BiOCl@IrO_2_–Ta_2_O_5_) recorded at scan rates of 5–30 mV s^−1^. With increasing scan rate, the magnitudes of the anodic and cathodic current densities increased, and the area enclosed by the CV curves expanded, while their overall profiles remained similar.

### 3.2. The Performance of Denitrification and Tetracycline Removal and the Influencing Factors

#### 3.2.1. The Performance of Denitrification and Tetracycline Removal

Nitrate electroreduction coupled with tetracycline removal was evaluated using the prepared BiOCl/DSA electrode or a commercial DSA electrode as the anode and ZVI/Co_3_O_4_/GF as the cathode. As shown in Figure 3, the DIN removal efficiencies with BiOCl/DSA and DSAs were 71.89% and 60.17%, respectively. The corresponding nitrate removal efficiencies were 99.61% and 99.55%, NH_4_^+^-N concentrations were 28.48 and 44.54 mg/L, NO_2_^−^-N concentrations were 0.08 and 0.01 mg/L, and TC removal efficiencies were 100% and 74.51%. Compared with the commercial DSA anode, the BiOCl/DSA system showed lower NH_4_^+^-N accumulation and higher apparent DIN removal than the DSA system. The lower NH_4_^+^-N accumulation and higher apparent DIN removal may be associated with chlorine-mediated oxidation, consistent with the higher free residual chlorine concentrations measured for BiOCl/DSA. In addition, TC was effectively removed in the BiOCl/DSA system, reducing the occupation of electrode active sites by organic molecules and alleviating competitive adsorption with NO_3_^−^, thereby favouring nitrate-reduction selectivity.

#### 3.2.2. Effect of Initial Nitrate-Nitrogen Concentration

Figure 4 shows the changes in nitrate-reduction products at different initial NO_3_^−^-N concentrations in the ZVI/Co_3_O_4_/GF cathode–BiOCl/DSA anode system at 1000 mg/L KCl. With an initial TC concentration of 10 mg/L and initial NO_3_^−^-N concentrations of 50, 150 and 200 mg/L, nitrate removal efficiencies after 160 min were 94.07%, 84.54% and 68.08%, respectively. The corresponding NO_2_^−^-N concentrations were 0.25, 2.78 and 7.07 mg/L; NH_4_^+^-N concentrations were 0.12, 47.55 and 30.72 mg/L; and DIN removal efficiencies were 69.91%, 51.97% and 50.03%. Increasing the initial nitrate-nitrogen concentration decreased nitrate and DIN removal but increased ammonium and nitrite formation. At higher nitrate loadings, saturation of electrode active sites and enhanced mass-transfer limitations likely lowered the reaction efficiency per unit time. Chloride addition increased apparent DIN removal and reduced NH_4_^+^-N accumulation, consistent with further transformation of cathodically generated NH_4_^+^.

As shown in Figure S3b, TC removal approached 100% at initial NO_3_^−^-N concentrations of 50, 150 and 200 mg/L, even at low chloride concentrations. However, the TC oxidation rate decreased slightly with increasing nitrate concentration, probably because nitrate competed with TC for electrode active sites, reduced TC adsorption and altered solution pH, thereby affecting the electrochemical oxidation rate of TC.

#### 3.2.3. Effect of Initial Tetracycline Concentration

Figure 5a–d presents nitrogen species during nitrate reduction in the ZVI/Co_3_O_4_/GF cathode–BiOCl/DSA anode system at 1000 mg/L KCl and different TC concentrations.

At an initial NO_3_^−^-N concentration of 100 mg/L and initial TC concentrations of 5, 10 and 20 mg/L, nitrate removal efficiencies after 160 min were 84.38%, 93.33% and 74.67%, respectively. The corresponding NO_2_^−^-N concentrations were 0.49, 0.55 and 0.58 mg/L; NH_4_^+^-N concentrations were 24.45, 14.87 and 27.94 mg/L; and DIN removal efficiencies were 60.22%, 78.32% and 49.42%. An appropriate TC concentration (10 mg/L) enhanced nitrate reduction and DIN removal while minimizing NH_4_^+^ accumulation, likely because TC oxidation at the anode and nitrate reduction at the cathode achieved a favourable electron distribution. At 20 mg/L TC, nitrate removal decreased and NH_4_^+^ formation increased, potentially because TC competed with NO_3_^−^ for electrode active sites or altered the local pH [23,24].

TC removal reached 100% within 160 min at all initial TC concentrations (Figure S3c). Nevertheless, the removal rate decreased with increasing initial TC concentration because more pollutant molecules were present while the number of active sites remained constant. Higher pollutant concentrations may also increase mass-transfer resistance and limit TC diffusion to the electrode surface.

#### 3.2.4. Effect of Initial pH

Figure 6a–d shows nitrate-reduction products in the ZVI/Co_3_O_4_/GF cathode–BiOCl/DSA anode system at 1000 mg/L KCl and different initial pH values. With an initial TC concentration of 10 mg/L and initial pH values of 5, 6 and 10, nitrate removal efficiencies after 160 min were 95.45%, 99.05% and 93.12%, respectively. The corresponding NO_2_^−^-N concentrations were 0.44, 0.25 and 0.31 mg/L, while NH_4_^+^-N concentrations were 10.15, 34.87 and 36.90 mg/L. DIN removal efficiencies were 86.76%, 65.94% and 58.30%, respectively. Nitrite accumulation was low, with a formation rate below 1%. The denitrification trends at pH 6 and 10 were nearly identical. Previous work [25] showed that NH_4_^+^ oxidation at pH < 6 mainly relies on chlorine radicals generated by anodic electrochlorination. At higher pH, the NH_4_^+^/NH_3_ equilibrium shifts toward free NH_3_, which reacts more readily with reactive chlorine species such as HOCl. Compared with Pt anodes, the BiOCl/DSA system showed relatively favourable nitrogen removal under alkaline conditions, possibly because alkaline media neutralize H^+^ generated during NH_4_^+^-N oxidation, retard conversion of free NH_3_ to NH_4_^+^ and thereby accelerate the oxidation pathway.

Figure S3d shows that TC removal reached 100% at all initial pH values because of chloride and the enhanced oxidation capacity of the BiOCl/DSA anode. At pH 5, nitrate and DIN removal were optimal and the TC degradation rate increased. The acidic environment likely favoured nitrate adsorption and reduction at the cathode surface and enhanced reactive-species generation, thereby promoting anodic TC oxidation.

#### 3.2.5. Effect of Cathode Potential

Figure 7a–d shows nitrate-reduction products in the ZVI/Co_3_O_4_/GF cathode–BiOCl/DSA anode system at 1000 mg/L KCl and different cathode potentials. With an initial TC concentration of 10 mg/L and an initial NO_3_^−^-N concentration of 100 mg/L, nitrate removal efficiencies after 160 min at −1.2, −1.4, −1.61 and −1.8 V versus Ag/AgCl were 70.89%, 100%, 92.83% and 97.15%, respectively. The corresponding NO_2_^−^-N concentrations were 0.52, 0.08, 0.46 and 0.04 mg/L; NH_4_^+^-N concentrations were 27.79, 0, 8.21 and 8.39 mg/L; and DIN removal efficiencies were 45.10%, 100%, 82.56% and 88.94%. The BiOCl/DSA system achieved approximately 100% apparent DIN removal at a cathode potential of −1.4 V versus Ag/AgCl. Shifting the cathode potential from −1.2 to −1.4 V versus Ag/AgCl increased cathodic electron supply and promoted nitrate reduction. Further shifting the cathode potential to more negative values did not further improve DIN removal under the tested conditions.

Figure S3e shows that TC removal reached 100% at all cathode potentials examined. TC removal increased with voltage because a larger electron supply accelerated interfacial electron transfer and pollutant degradation. The improvement became negligible from −1.61 to −1.8 V versus Ag/AgCl, indicating that excessive electrons may occupy active sites and hinder TC removal, consistent with the nitrate-reduction results.

#### 3.2.6. Effect of Initial Chloride Concentration

The BiOCl/DSA anode exhibited a greater capacity for removing cathodically generated NH_4_^+^-N than the unmodified DSA anode, suggesting that Cl^−^ plays an important role in regulating nitrogen transformation in this system. To further determine the effect of chloride concentration, nitrate electroreduction and nitrogen-product distributions were examined at initial KCl concentrations of 50, 100, 300, 1000, 1500, and 2000 mg/L, with the cathode potential maintained at −1.61 V versus Ag/AgCl and the initial pH at 7 (Figure 8). Across the investigated KCl concentration range, NO_3_^−^-N removal remained high, ranging from 82.46% to 100%. However, NO_3_^−^-N removal decreased slightly when the KCl concentration exceeded 1000 mg/L. By comparison, DIN removal was more sensitive to KCl concentration, increasing from 56.00% to a maximum of 100% and subsequently decreasing to 79.60% at higher KCl concentrations.

At KCl concentrations of 50, 100, 300, 1000, 1500, and 2000 mg/L, the final NO_2_^−^-N concentrations were 0.43, 0.17, 0.35, 0.015, 0.44, and 0.46 mg/L, respectively. All values remained below 1 mg/L, indicating negligible NO_2_^−^-N accumulation under the investigated conditions. The corresponding residual NH_4_^+^-N concentrations were 45.07, 35.34, 23.79, 0, 10.15, and 2.45 mg/L, respectively. Residual NH_4_^+^-N generally decreased as the KCl concentration increased from 50 to 1000 mg/L and fell below the detection limit at 1000 mg/L KCl. At higher KCl concentrations, NH_4_^+^-N exhibited a nonmonotonic response. Thus, NH_4_^+^-N accumulation did not vary monotonically with chloride concentration but reflected a dynamic balance between its production through cathodic NO_3_^−^-N reduction and its oxidative consumption at the anode. An appropriate chloride supply may enhance anodic ClER and reactive-chlorine generation, thereby promoting the subsequent oxidation of cathodically generated NH_4_^+^-N and reducing the accumulation of dissolved nitrogen intermediates.

At an initial NO_3_^−^-N concentration of 100 mg/L, an initial TC concentration of 10 mg/L, pH 7, and a cathode potential of −1.61 V versus Ag/AgCl, the highest calculated DIN removal efficiency of 100% was obtained at 1000 mg/L KCl. Under these conditions, NH_4_^+^-N was below the detection limit and the residual NO_2_^−^-N concentration was only 0.015 mg/L. Together with the anode-comparison results, these findings show that the BiOCl/DSA anode achieved greater DIN removal under moderate chloride supply, consistent with the proposed enhancement of ClER and reactive-chlorine generation by BiOCl modification.

Chloride concentration also affected TC removal (Figure S3f). At initial KCl concentrations of 50, 100, 300, 1000, 1500, and 2000 mg/L, the TC removal efficiencies were 91.85%, 100%, 99.99%, 100%, 100%, and 100%, respectively. Increasing the KCl concentration from 50 to 100 mg/L increased TC removal from 91.85% to approximately 100%, whereas further increases in KCl concentration produced no appreciable improvement in the final removal efficiency. These results suggest that an appropriate chloride supply promotes TC removal, likely through the participation of anodically generated reactive chlorine species in the indirect oxidation of TC.

### 3.3. Mechanism of Chloride Activation and Enhanced Denitrification over the BiOCl/DSA Anode

The ability of DSA and BiOCl/DSAs to generate reactive chlorine was compared at initial KCl concentrations listed in Table 1. Free residual chlorine concentrations were measured after 160 min, and the results are shown in Table 1. Free chlorine concentrations generated by BiOCl/DSA were consistently higher than those generated by the unmodified DSA, particularly at low chloride concentrations, indicating stronger chlorine-evolution capability. Combined residual chlorine was also detected in both systems at the tested KCl concentrations (Table 1).

To further clarify the difference between the two anodes, linear sweep voltammetry (LSV) curves were measured for BiOCl/DSA and DSA in electrolytes with different chloride concentrations. As shown in Figure 9a–c, the polarization curves of both electrodes shifted with chloride concentration: higher chloride concentrations produced progressively more negative chlorine-evolution onset potentials and markedly higher current densities. This behaviour is consistent with the Nernst equation for chlorine evolution. Compared with DSA, the BiOCl/DSA anode exhibited a lower onset potential and a higher current response at the same chloride concentration. Chlorine evolution on DSA is generally considered to follow a Volmer–Heyrovský mechanism (Equations (1) and (2)) [26,27], comprising electrochemical adsorption of Cl^−^ at an active site (S) to form an adsorbed intermediate (S–Cl•) in the Volmer step, followed by reaction of adsorbed Cl• with solution-phase Cl^−^ to form Cl_2_ and desorb from the electrode surface in the Heyrovský step.

(1) $$S+{\mathrm{Cl}}^{-}\;⇄\;S\text{–}\mathrm{Cl}(\mathrm{ads})+e^{-}$$

(2) $$S\text{–}\mathrm{Cl}(\mathrm{ads})+{\mathrm{Cl}}^{-}\;\to \;S+{\mathrm{Cl}}_{2}+e^{-}$$

To investigate the possible involvement of reactive species in TC degradation, quenching experiments were conducted using TBA, KBrO_3_, and EDTA (Figure S4). The addition of TBA markedly inhibited TC degradation, with C/C_0_ remaining at approximately 0.50 after 160 min, whereas KBrO_3_ and EDTA showed no comparable inhibitory effects. These results suggest that TBA-reactive short-lived radical species participated in TC degradation. Because TBA is not selective for Cl• in chloride-containing systems, the results provide indirect evidence for radical involvement but do not directly identify Cl• as the dominant reactive species.

The improved denitrification performance of the BiOCl/DSA anode may be associated with its enhanced chlorine-evolution behaviour. Although the BiOCl layer has relatively poor electronic conductivity, the underlying mixed-metal-oxide DSA exhibits semiconducting properties and can form a stable ohmic contact between the BiOCl layer and the conductive substrate. This architecture promotes strong interfacial interactions and charge redistribution [21]. The Ru–Ir–Ti DSA anode also has favourable selective chlorine-evolution activity and electrocatalytic performance [28,29]. Competitive OER may occur at DSAs and affect the Faradaic efficiency of in situ chlorine generation [30,31,32]. The BiOCl/DSA anode produced higher free-chlorine concentrations than the unmodified DSA anode under the tested conditions.

At the anode interface, chloride may undergo electrochemical oxidation [33,34] to adsorbed chlorine intermediates, followed by the formation of Cl_2_ and other reactive chlorine species. Literature-reported solution reactions involving Cl•, Cl_2_•^−^, Cl_2_, and HOCl may subsequently occur (Equations (3)–(6)) [35]. These reactive chlorine species can diffuse into solution and participate in subsequent oxidation reactions. In addition, chloride-deficient sites in BiOCl, such as BiOCl_1−x_(OH)_x_, have strong affinity for solution Cl^−^ (the pKsp of BiOCl is 30.75), enabling rapid chloride replenishment (Equation (7)) [36]. This chloride-replenishment process may facilitate continuous interfacial chloride activation.

In the proposed BiOCl/DSA design, the underlying DSA layer provides an efficient electron-transport pathway from the anode to the outer interface. The outer BiOCl layer regulates interfacial reaction selectivity and may facilitate interfacial chloride adsorption and activation, thereby promoting chlorine evolution [37] and increasing the yield of reactive chlorine species. These species may contribute to the further transformation of cathodically generated NH_4_^+^, consistent with the observed increase in DIN removal. A schematic illustration of the proposed mechanism for synergistic electrochemical denitrification and tetracycline degradation is presented in Figure 10.

(3) $${\mathrm{Cl}}^{\cdot }+{\mathrm{Cl}}^{-}\to {\mathrm{Cl}}_{2}^{\cdot -}$$

(4) $${\mathrm{Cl}}_{2}^{\cdot -}+{\mathrm{Cl}}_{2}^{\cdot -}\to {\mathrm{Cl}}_{2}+2{\mathrm{Cl}}^{-}$$

(5) $${\mathrm{Cl}}_{2}^{\cdot -}+{\mathrm{HO}}^{\cdot }\to \mathrm{HClO}+{\mathrm{Cl}}^{-}$$

(6) $${\mathrm{Cl}}_{2}+H_{2}O⇄{\mathrm{HClO}+H}^{+}+{\mathrm{Cl}}^{-}$$

(7) $${\mathrm{BiOCl}}_{1-x}{\left(\mathrm{OH}\right)}_{X}+{\mathrm{xCl}}^{-}\to {\mathrm{BiOCl}+\mathrm{xOH}}^{-}$$

Previous work showed that BiOCl functionalization enhanced chlorine generation and ammonium oxidation on mixed-metal-oxide anodes, with mechanistic evidence linking these effects to chloride adsorption and chlorine-radical formation [21]. A Volmer–Heyrovský mechanism has also been reported for chlorine evolution on RuO_2_ and IrO_2_ electrodes [38]. These findings provide a basis for considering chlorine-mediated oxidation in the present system. The higher free residual chlorine concentrations and lower NH_4_^+^-N accumulation observed with BiOCl/DSA suggest that reactive chlorine species may contribute to the subsequent oxidation of cathodically generated NH_4_^+^. This process may help explain the higher apparent DIN removal achieved with BiOCl/DSA and may be beneficial in systems where ammonium accumulation is of concern. The preceding analysis is based mainly on single-factor experiments; chloride concentration, cathode-potential magnitude and pH may jointly influence NH_4_^+^-N formation and consumption, motivating further analysis of their combined effects using a multivariable model.

### 3.4. Data-Driven Prediction and Parameter-Response Analysis of NH_4_^+^-N

#### 3.4.1. Model Comparison and Optimization for NH_4_^+^-N Concentration Prediction

To characterize the response of NH_4_^+^-N concentration to multiple operating parameters, FNN, BP, GA-BP, and PSO-BP models were constructed and compared. Because chloride concentration, cathode-potential magnitude, and pH may produce nonlinear changes in NH_4_^+^-N formation and consumption, a multivariable model was used to describe the relationship between the investigated operating parameters and NH_4_^+^-N concentration.

The predictive performances of FNN, BP, GA-BP and PSO-BP for NH_4_^+^-N concentration were compared to identify the optimal model for this system [39,40]. As shown in Table 2, FNN exhibited higher accuracy and stability than the other models on both the training and test sets. On the test set, the MAE, MSE, RMSE and R^2^ values of the FNN model were 0.3560, 0.3186, 0.5645 and 0.9507, respectively, all superior to those of BP, GA-BP and PSO-BP.

Compared with conventional BP, FNN achieved higher accuracy for NH_4_^+^-N concentration prediction within the current dataset. As shown in Figure 11, BP predictions were relatively dispersed, whereas GA-BP and PSO-BP reduced the deviations between the predicted and measured values. FNN predictions showed the closest agreement with the measured values and the ideal prediction line, indicating that FNN more effectively represented the nonlinear variation in NH_4_^+^-N concentration within the investigated data range. FNN was therefore selected for the subsequent parameter-response analysis. Nevertheless, given the limited dataset, its predictive performance for new operating conditions requires further validation using independent data.

#### 3.4.2. FNN-Based Analysis of Multivariable Response Trends

The trained FNN model was used to analyze the predicted responses of NH_4_^+^-N formation and consumption to changes in operating parameters. Inputs were reaction time (0–160 min), KCl concentration, initial NO_3_^−^-N concentration (50–200 mg/L), cathode-potential magnitude (1.2–1.8 V versus Ag/AgCl) and pH (5–10), and the output was NH_4_^+^-N concentration. At a fixed reaction time of 160 min, KCl concentration, voltage, pH and initial NO_3_^−^-N concentration were varied to construct model-predicted multivariable response maps using MATLAB R2022_b_ (MathWorks, Natick, MA, USA) (Figure 12). These response maps represent model-predicted trends rather than direct experimental validation of interactions among the operating parameters.

FNN predictions showed that NH_4_^+^-N formation and accumulation do not vary linearly with a single operating parameter. The predicted patterns are consistent with a balance between cathodic NO_3_^−^-N reduction to NH_4_^+^-N and the subsequent anodic consumption of NH_4_^+^-N. Figure 12 shows pronounced nonlinear responses of NH_4_^+^-N concentration, indicating that single-factor analysis cannot fully characterize the dynamic nitrogen-conversion pathways in this system. Chloride concentration and voltage primarily affect anodic ClER and the generation intensity of reactive chlorine species, whereas pH changes nitrogen-conversion pathways by altering the NH_4_^+^/NH_3_ equilibrium and reactive-chlorine speciation. NH_4_^+^-N accumulation should therefore be understood as the dynamic balance between its formation rate and anodic oxidative-consumption rate rather than being attributed solely to nitrate-reduction strength.

The distribution of FNN-predicted NH_4_^+^-N concentration was further analyzed. Figure S5 shows pronounced nonlinear responses at different initial NO_3_^−^-N concentrations and pH values, indicating that the model-predicted NH_4_^+^-N accumulation varies with KCl concentration, voltage, pH, and initial NO_3_^−^-N concentration. Because pH 7 is close to neutral nitrogen-containing wastewater, the predicted results at pH 7 were further analyzed using the NH_4_^+^-N yield. Figure 13 shows that high generation-rate regions were mainly located at low KCl concentrations, especially at approximately 50–200 mg/L KCl and 1.2–1.5 V versus Ag/AgCl, where relative NH_4_^+^-N accumulation was more likely. Increasing KCl concentration generally decreased the NH_4_^+^-N yield, indicating strengthened oxidative consumption of NH_4_^+^-N. The generation rate did not increase monotonically with influent nitrate concentration. At initial NO_3_^−^-N concentrations of 50, 100, 150 and 200 mg/L, the maximum NH_4_^+^-N yield at pH 7 were 53.09%, 69.41%, 77.64% and 57.42%, respectively. Relative ammonium accumulation was most pronounced at 150 mg/L NO_3_^−^-N. Although the predicted absolute NH_4_^+^-N concentration was higher at 200 mg/L, the generation fraction decreased, indicating that a higher substrate concentration does not necessarily produce stronger relative NH_4_^+^-N accumulation.

The FNN output predicted greater NH_4_^+^-N accumulation at relatively low KCl concentrations. Higher KCl concentrations were associated with lower model-predicted NH_4_^+^-N accumulation. Together with the single-factor effects of KCl concentration, voltage and pH on DIN removal and NH_4_^+^-N accumulation, the parameter region around 1000 mg/L KCl and −1.4 V versus Ag/AgCl was more favourable for reducing residual NH_4_^+^-N while maintaining NO_3_^−^-N reduction. The FNN model can therefore predict NH_4_^+^-N concentration and, through yield analysis, identify relative trends in nitrogen-conversion pathways at different influent nitrate concentrations, providing preliminary information for screening KCl concentration, voltage, and pH conditions. The present FNN model is based on predictions and parameter-response analysis within the existing experimental range; its extrapolation capability should be further validated using larger datasets and continuous-flow systems.

## 4. Conclusions

A BiOCl/DSA anode was self-assembled on a commercial DSA anode and applied to chloride-enhanced electrochemical denitrification coupled with tetracycline oxidation. Compared with the commercial DSA anode, the BiOCl/DSA anode maintained comparable nitrate-removal performance, increased DIN removal from 60.17% to 71.89%, decreased NH_4_^+^-N accumulation from 44.54 to 28.48 mg/L, and increased TC removal from 74.51% to 100%. Single-factor experiments showed that 50 mg/L initial NO_3_^−^-N, 10 mg/L initial TC, pH 5, a cathode potential of −1.4 V versus Ag/AgCl, and 1000 mg/L KCl were the respective favourable levels within the tested ranges when each factor was evaluated independently. The observed nitrogen-species changes suggest that nitrate reduction was accompanied by subsequent NH_4_^+^-N transformation, to which chlorine-mediated oxidation may contribute. The BiOCl/DSA system showed higher free residual chlorine concentrations than DSA under the tested KCl concentrations. Among FNN, BP, GA-BP and PSO-BP, FNN delivered the best NH_4_^+^-N prediction performance, with a test-set R^2^ of 0.9507. FNN response maps illustrated the predicted nonlinear variation in NH_4_^+^-N formation and consumption across the investigated ranges of reaction time, KCl concentration, initial NO_3_^−^-N concentration, voltage, and pH. The parameter region around 1000 mg/L KCl and a cathode potential of −1.4 V versus Ag/AgCl was associated with lower predicted NH_4_^+^-N accumulation, whereas approximately 100 mg/L KCl and −1.4 V versus Ag/AgCl resulted in high ammonium accumulation. This work provides experimental evidence and preliminary data-driven information for parameter screening in chloride-assisted electrochemical denitrification and TC oxidation using a BiOCl/DSA anode.

## Figures and Tables

**Figure 1:**
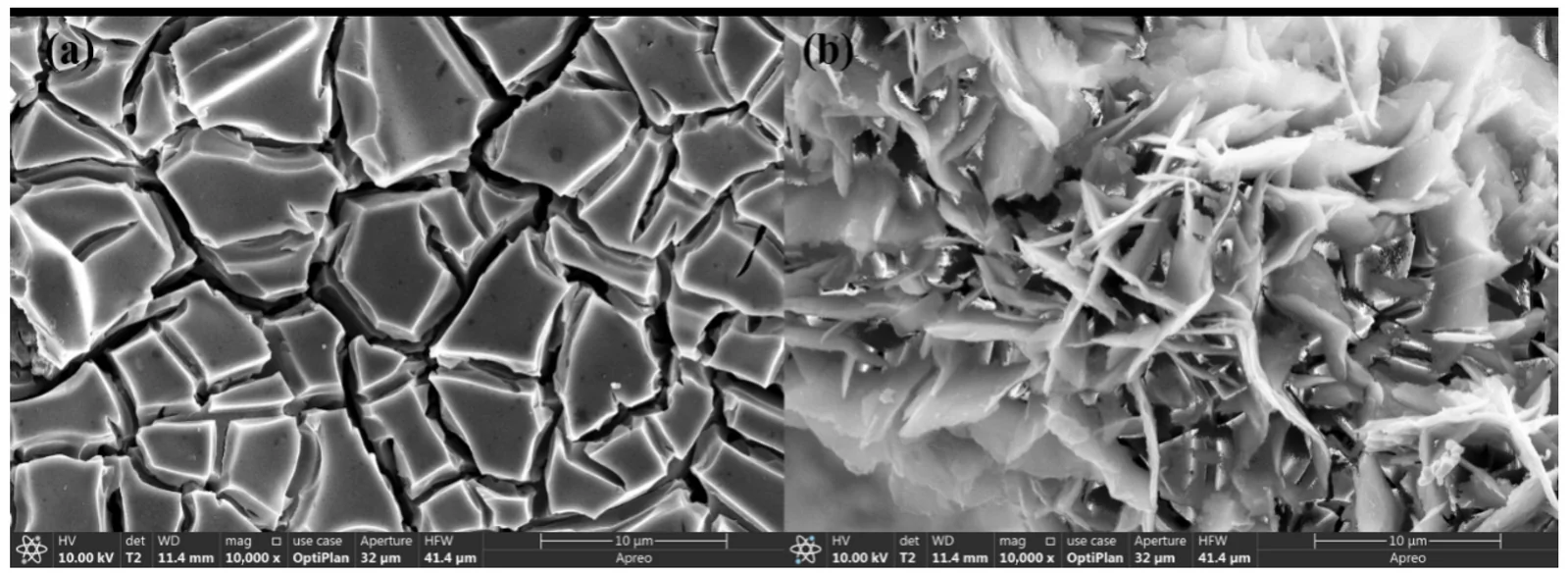
SEM images of (**a**) the DSA anode and (**b**) the self-assembled BiOCl/DSA anode.

**Figure 2:**
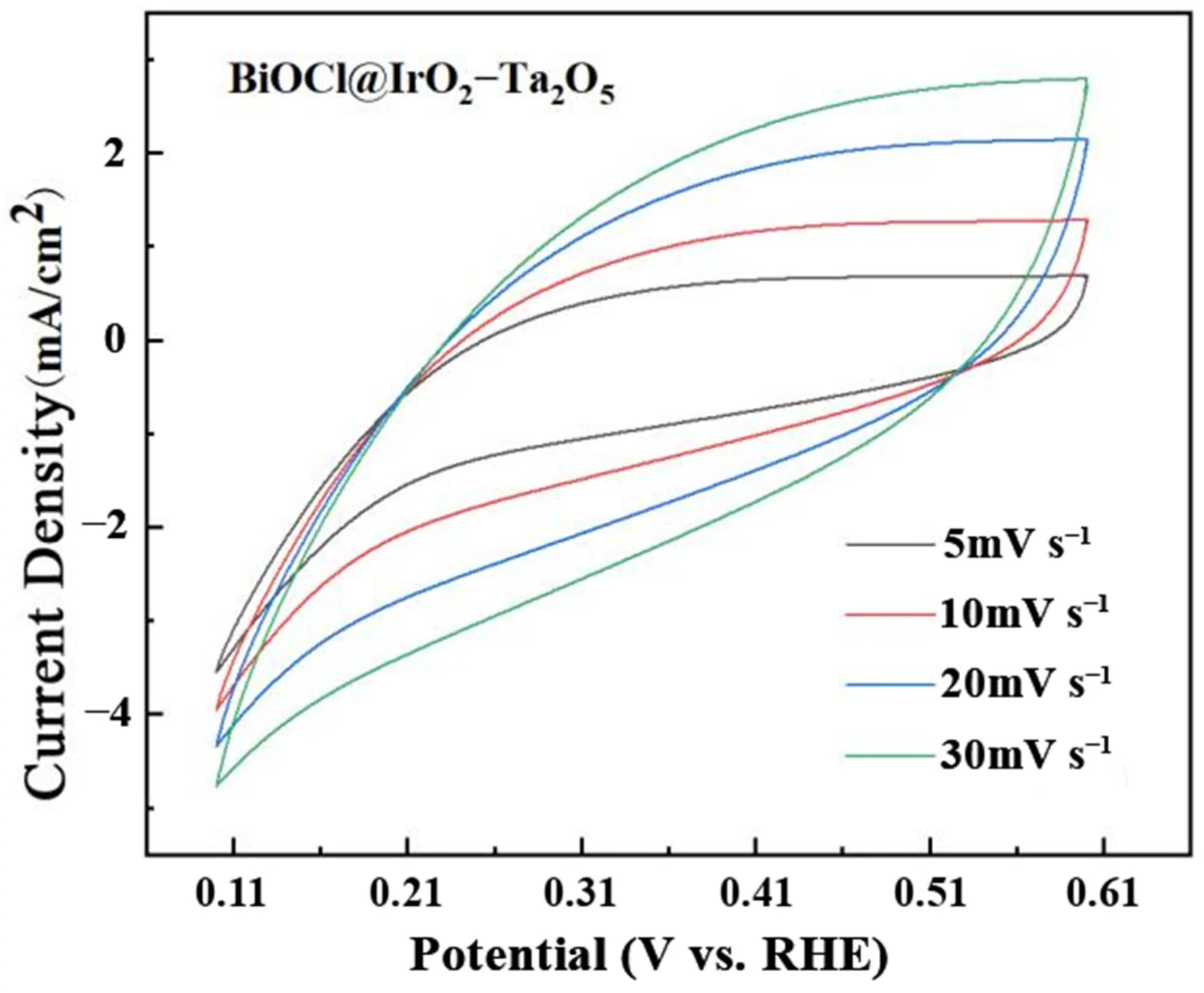
CV curves of the BiOCl/DSA anode at different scan rates.

**Figure 3:**
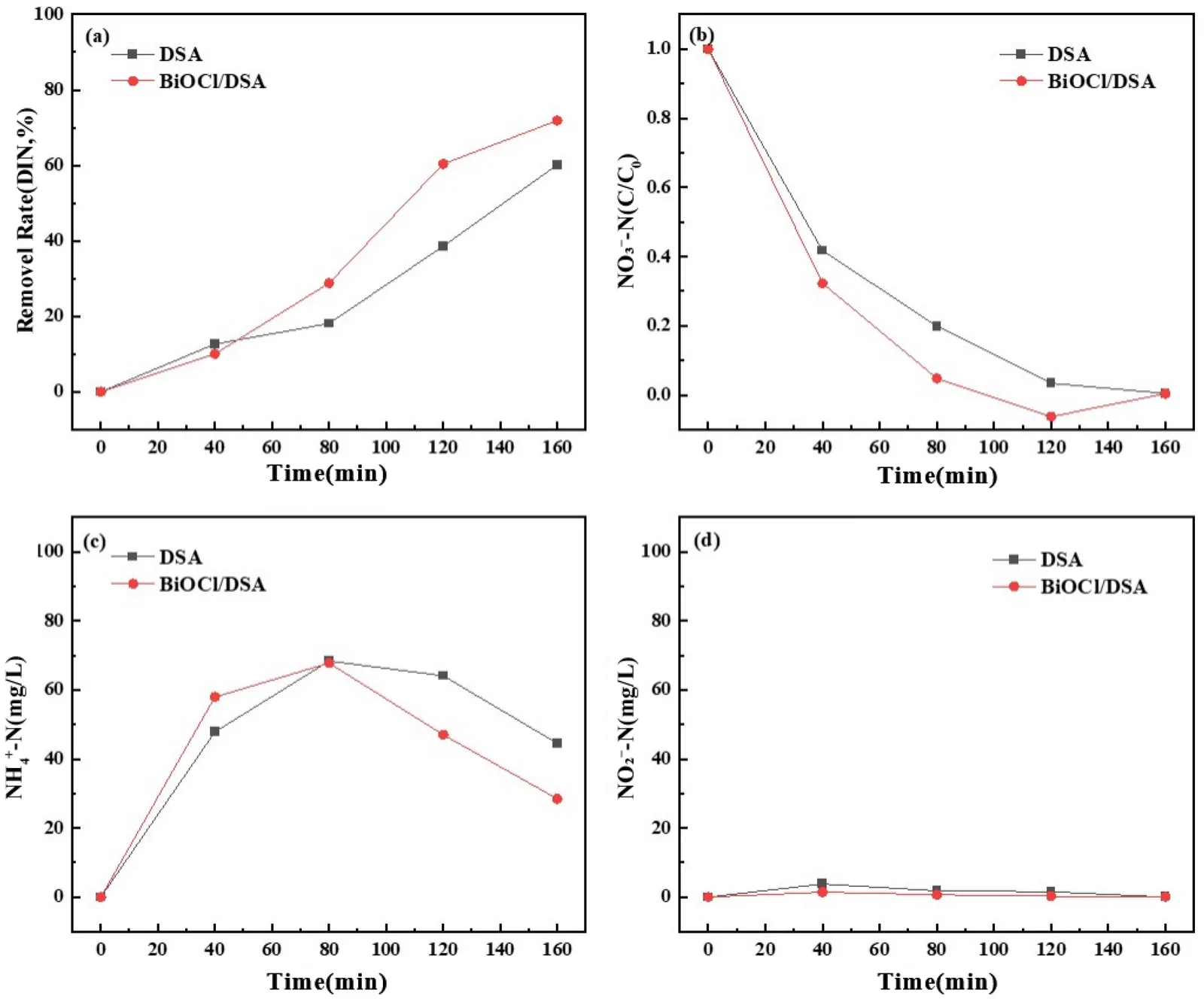
Nitrogen species with different anodes: (**a**) DIN removal efficiency, (**b**) NO_3_^−^-N removal, (**c**) NH_4_^+^-N formation and (**d**) NO_2_^−^-N formation (initial NO_3_^−^-N concentration, 100 mg/L; Na_2_SO_4_ concentration, 0.5 M; pH 7; cathode potential, −1.61 V versus Ag/AgCl).

**Figure 4:**
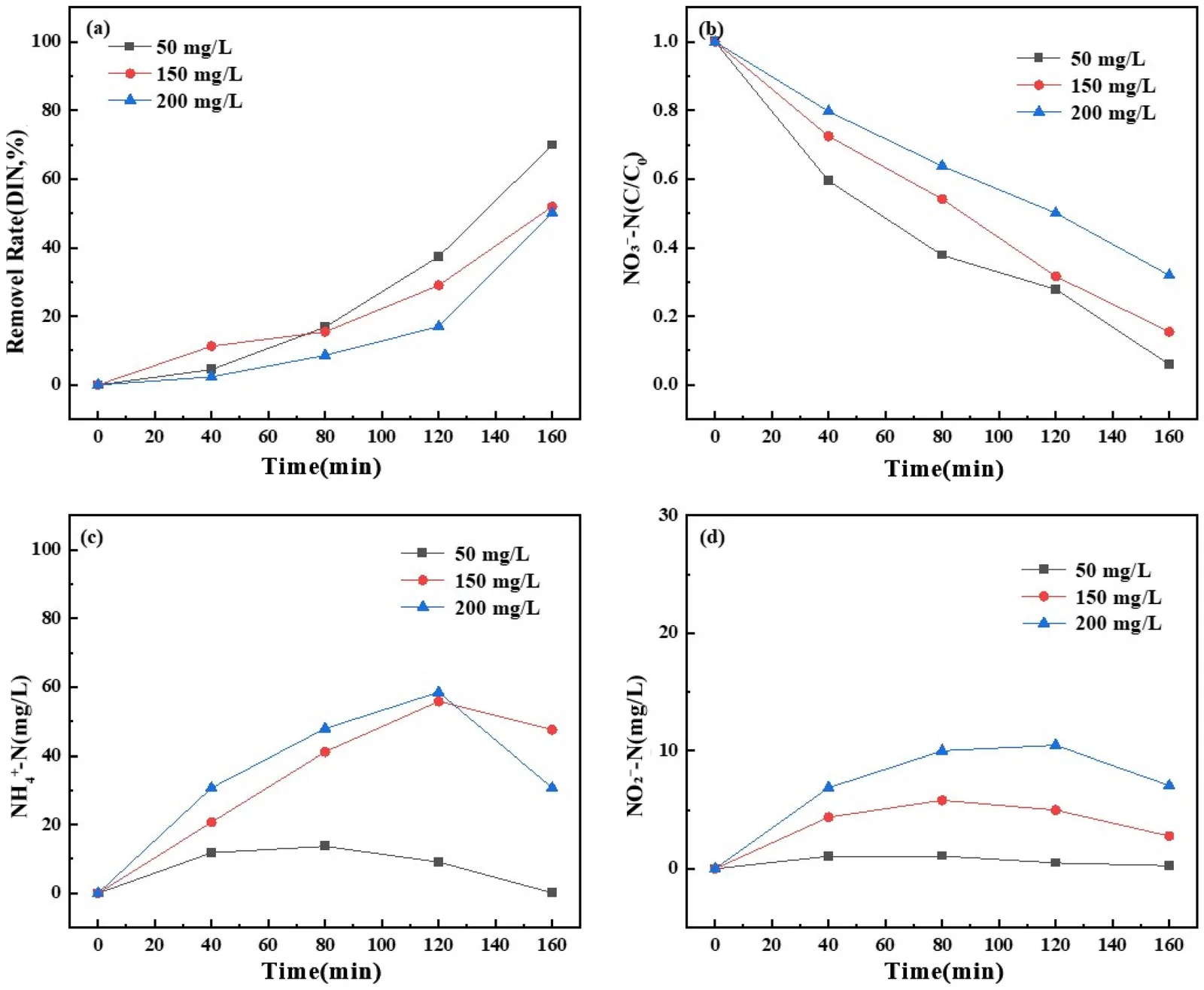
Denitrification at different initial NO_3_^−^-N concentrations: (**a**) DIN removal efficiency, (**b**) NO_3_^−^-N removal, (**c**) NH_4_^+^-N formation and (**d**) NO_2_^−^-N formation (initial TC concentration, 10 mg/L; Na_2_SO_4_ concentration, 0.5 M; pH 7; cathode potential, −1.61 V versus Ag/AgCl).

**Figure 5:**
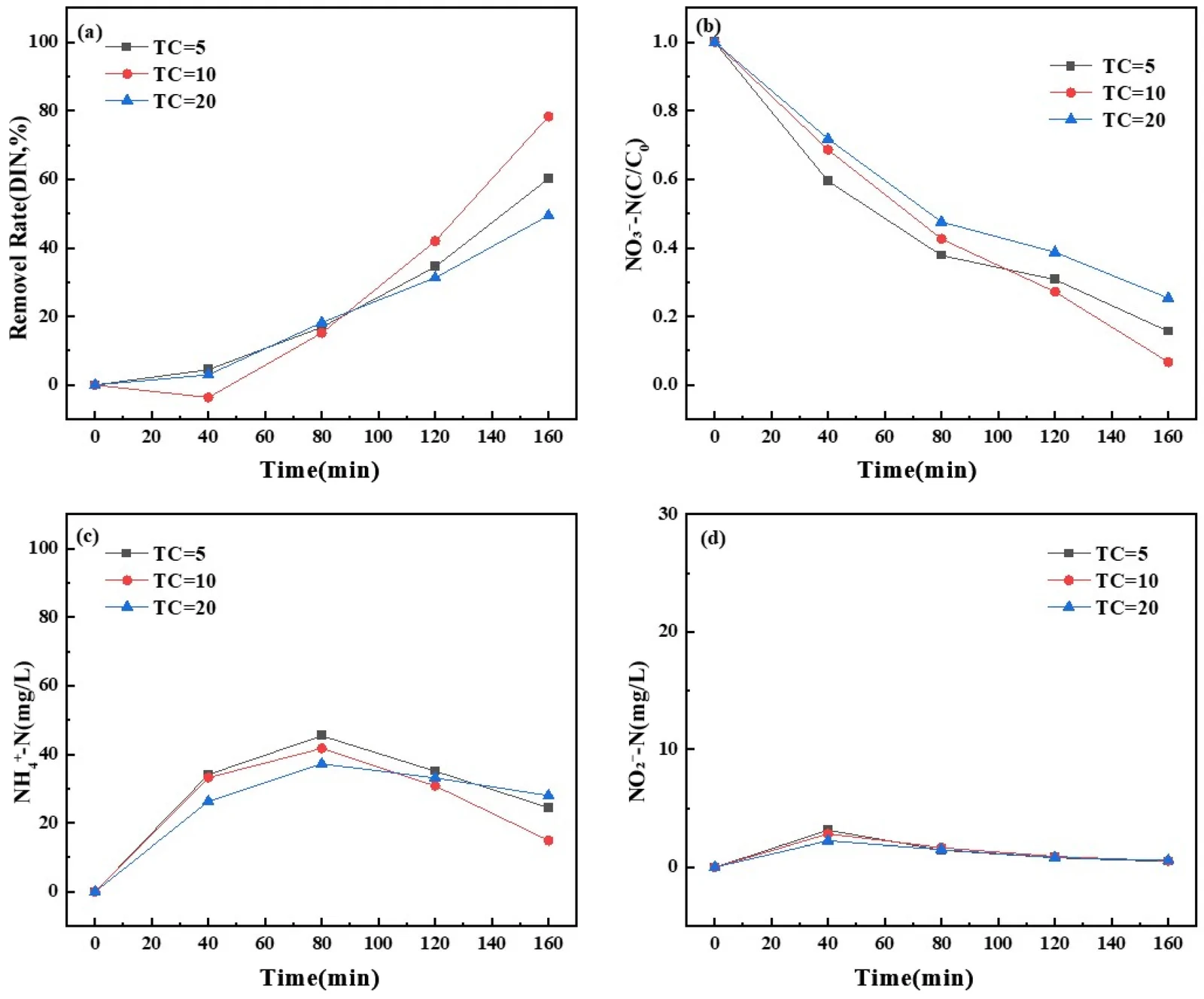
Denitrification at different initial TC concentrations: (**a**) DIN removal efficiency, (**b**) NO_3_^−^-N removal, (**c**) NH_4_^+^-N formation and (**d**) NO_2_^−^-N formation (initial NO_3_^−^-N concentration, 100 mg/L; Na_2_SO_4_ concentration, 0.5 M; pH 7; cathode potential, −1.61 V versus Ag/AgCl).

**Figure 6:**
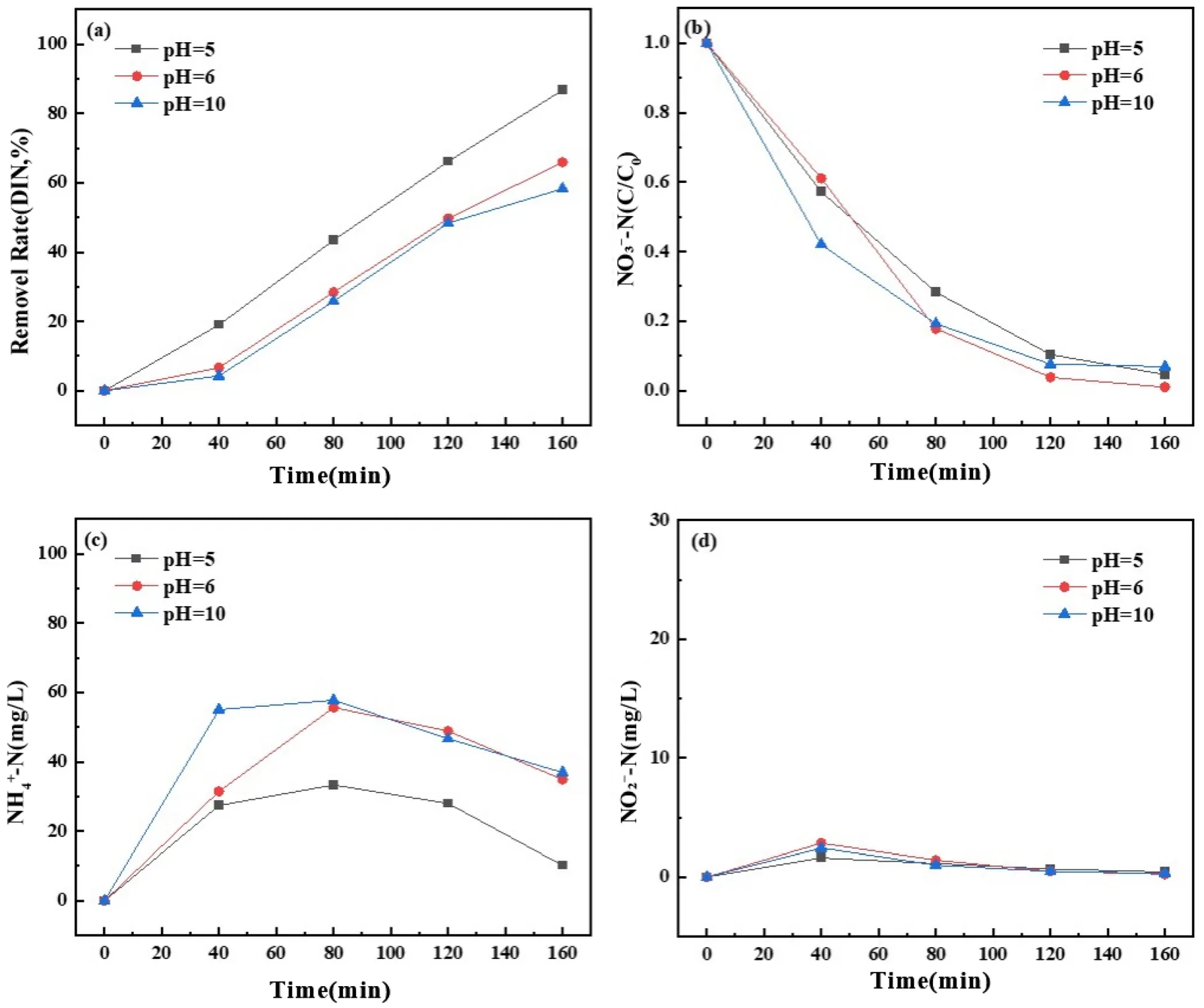
Denitrification at different initial pH values: (**a**) DIN removal efficiency, (**b**) NO_3_^−^-N removal, (**c**) NH_4_^+^-N formation and (**d**) NO_2_^−^-N formation (initial NO_3_^−^-N concentration, 100 mg/L; initial TC concentration, 10 mg/L; Na_2_SO_4_ concentration, 0.5 M; cathode potential, −1.61 V versus Ag/AgCl).

**Figure 7:**
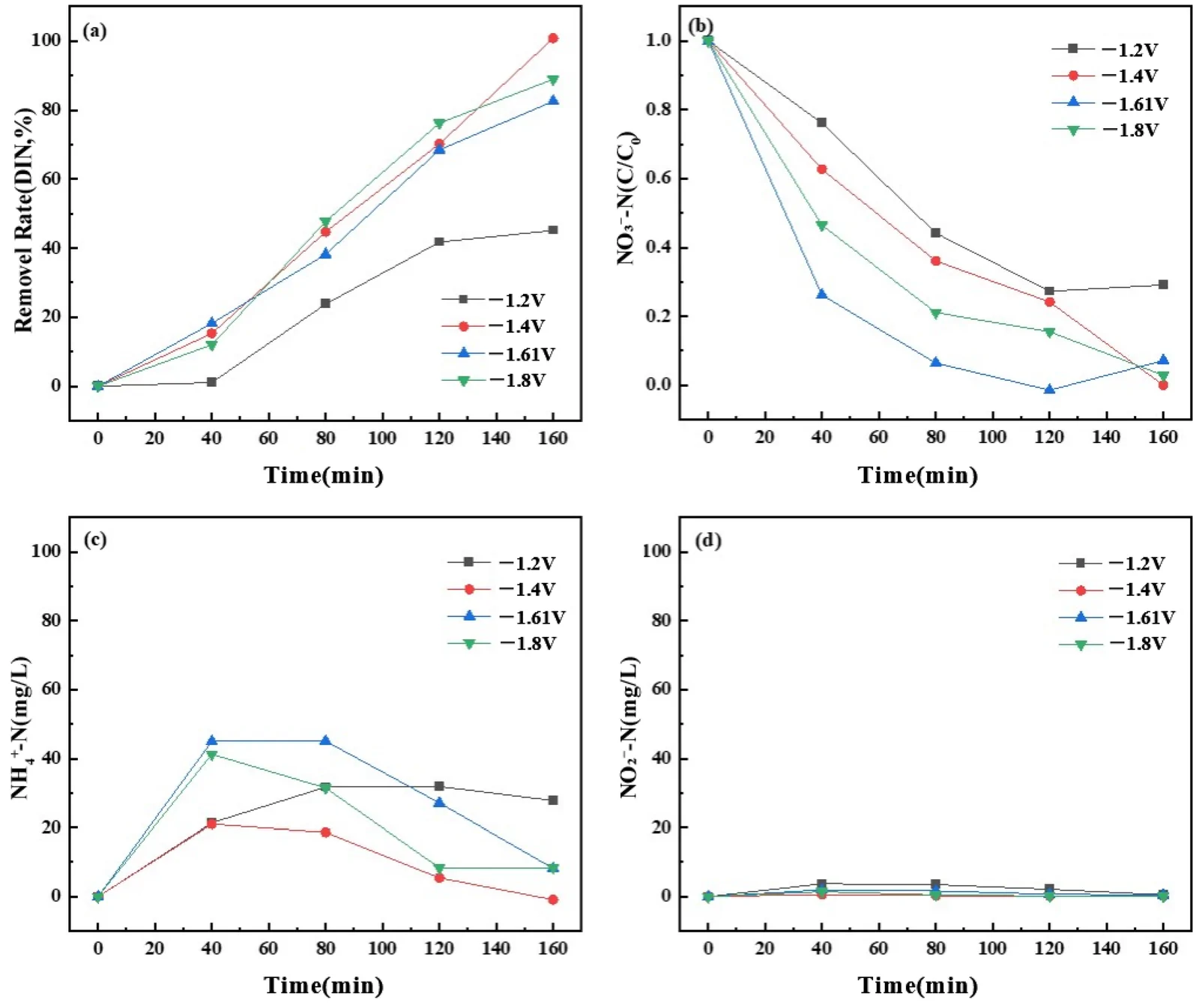
Denitrification at different cathode potentials: (**a**) DIN removal efficiency, (**b**) NO_3_^−^-N removal, (**c**) NH_4_^+^-N formation and (**d**) NO_2_^−^-N formation (initial NO_3_^−^-N concentration, 100 mg/L; initial TC concentration, 10 mg/L; Na_2_SO_4_ concentration, 0.5 M; pH 7).

**Figure 8:**
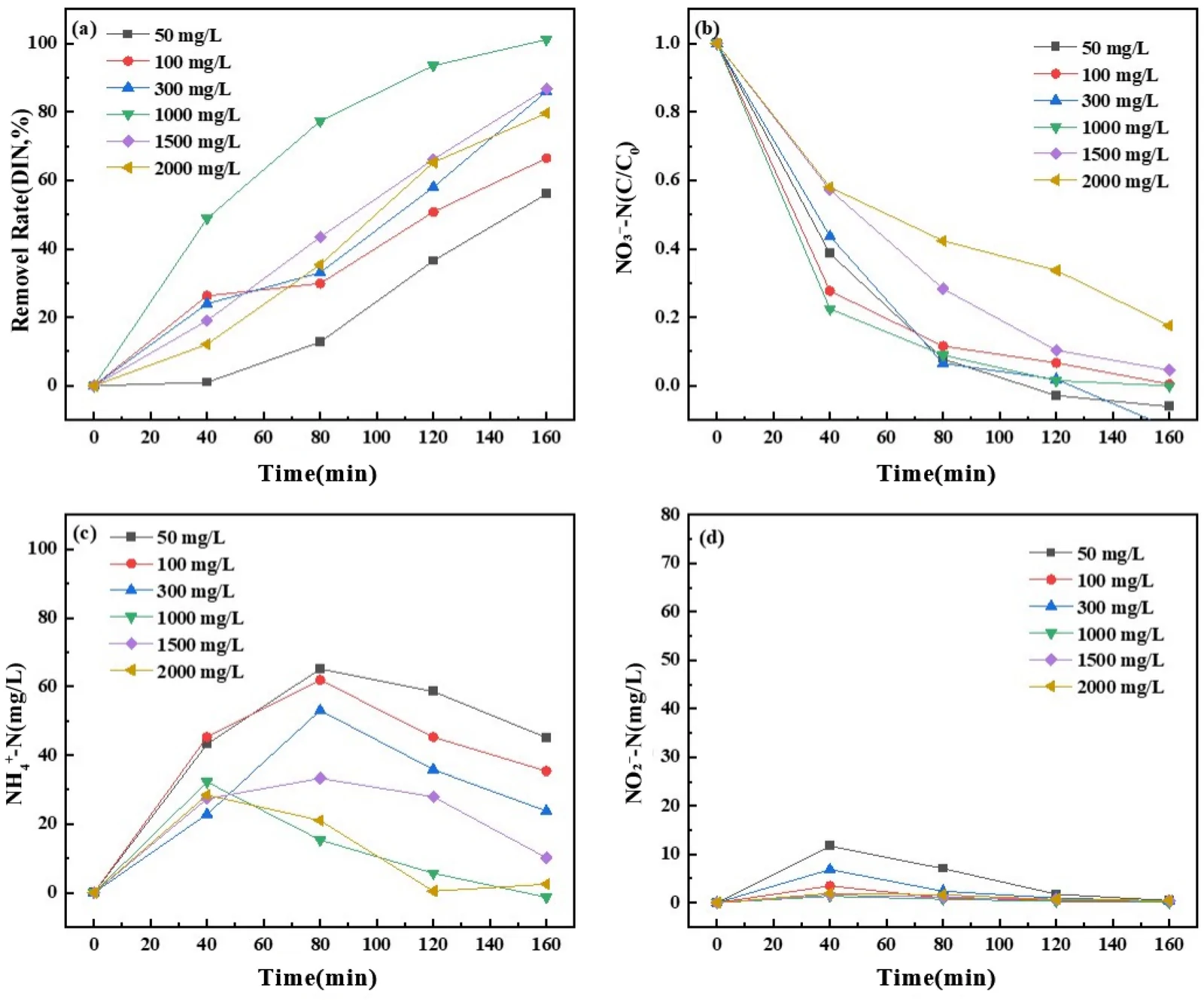
Denitrification at different KCl concentrations: (**a**) DIN removal efficiency, (**b**) NO_3_^−^-N removal, (**c**) NH_4_^+^-N formation and (**d**) NO_2_^−^-N formation (initial NO_3_^−^-N concentration, 100 mg/L; initial TC concentration, 10 mg/L; Na_2_SO_4_ concentration, 0.5 M; pH 7; cathode potential, −1.61 V versus Ag/AgCl).

**Figure 9:**
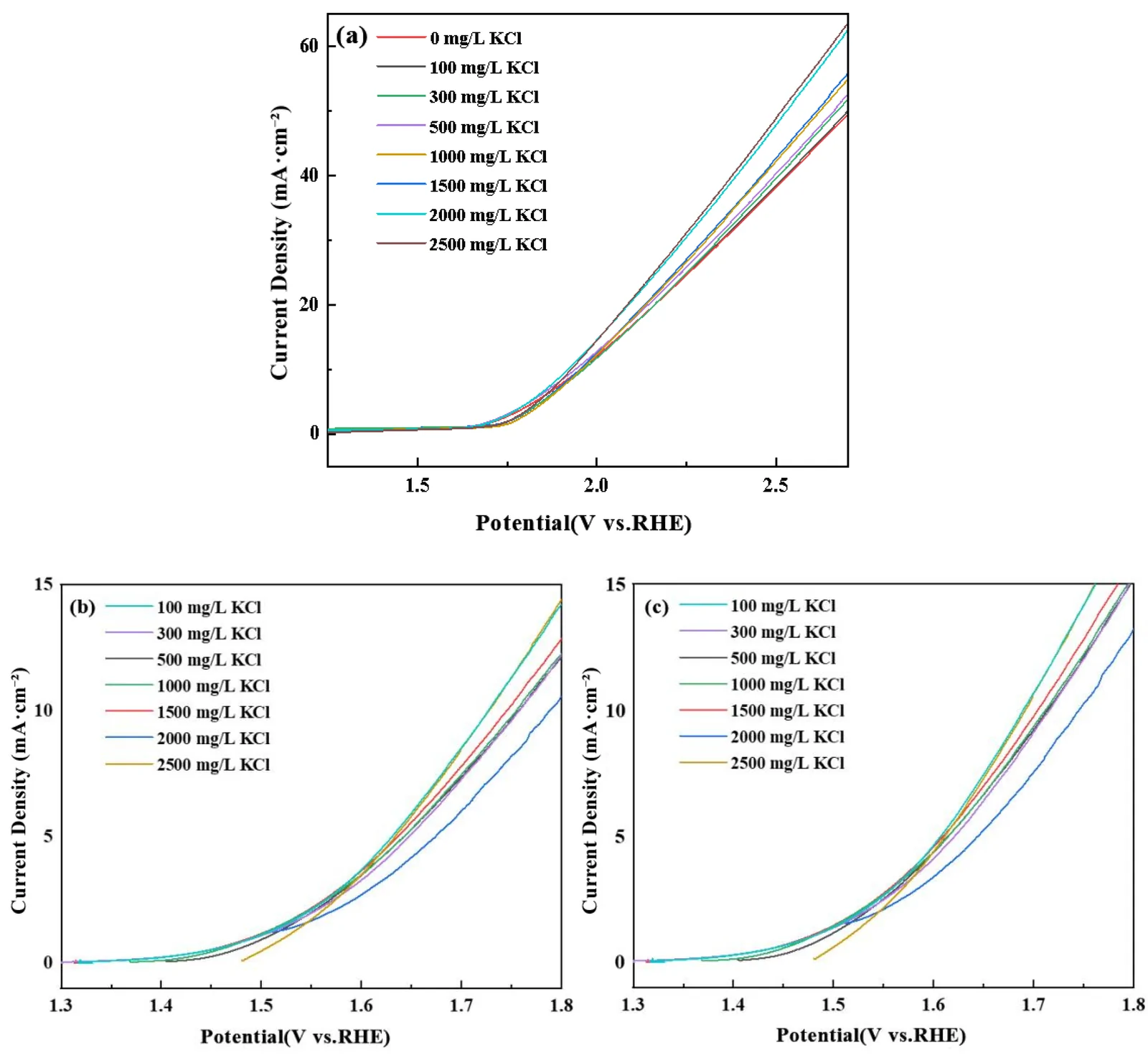
LSV curves of BiOCl/DSA at different KCl concentrations (**a**) and ClER polarization curves of DSA (**b**) and BiOCl/DSA (**c**) electrodes.

**Figure 10:**
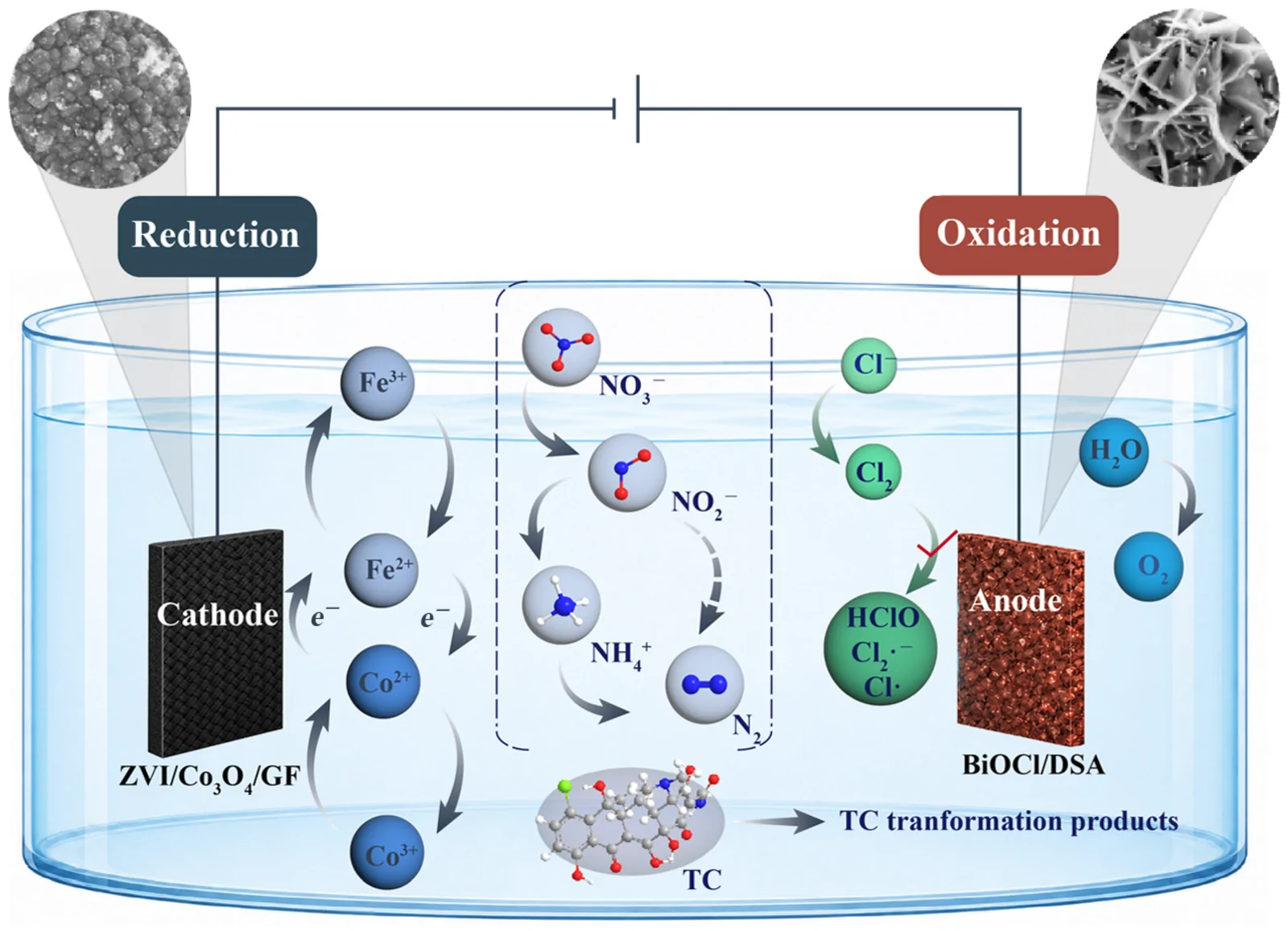
Proposed reaction pathways for nitrate conversion and tetracycline degradation in the electrochemical system.

**Figure 11:**
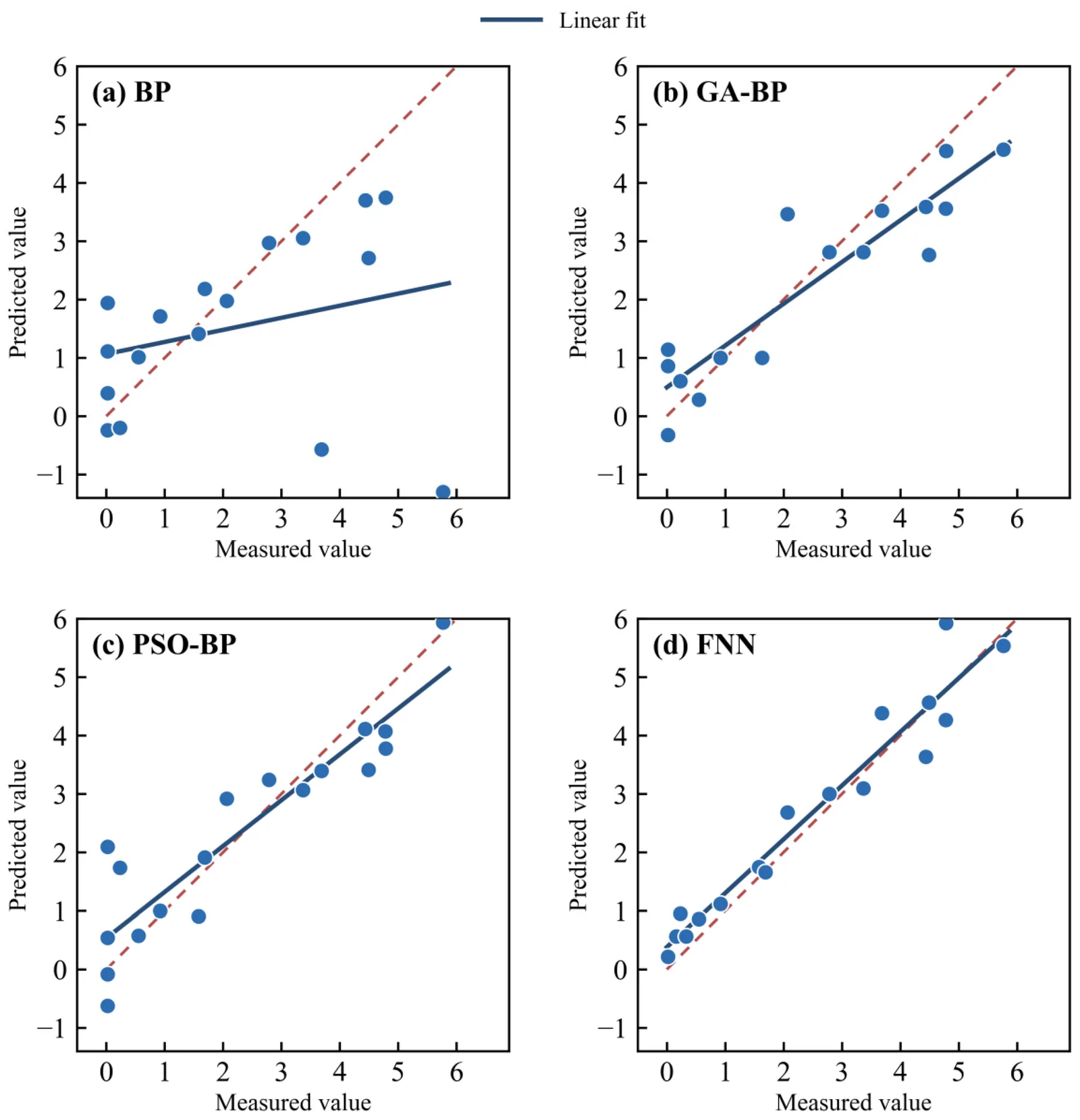
Regression comparison of predicted and measured NH_4_^+^-N concentrations for BP (**a**), GA-BP (**b**), PSO-BP (**c**) and FNN (**d**).

**Figure 12:**
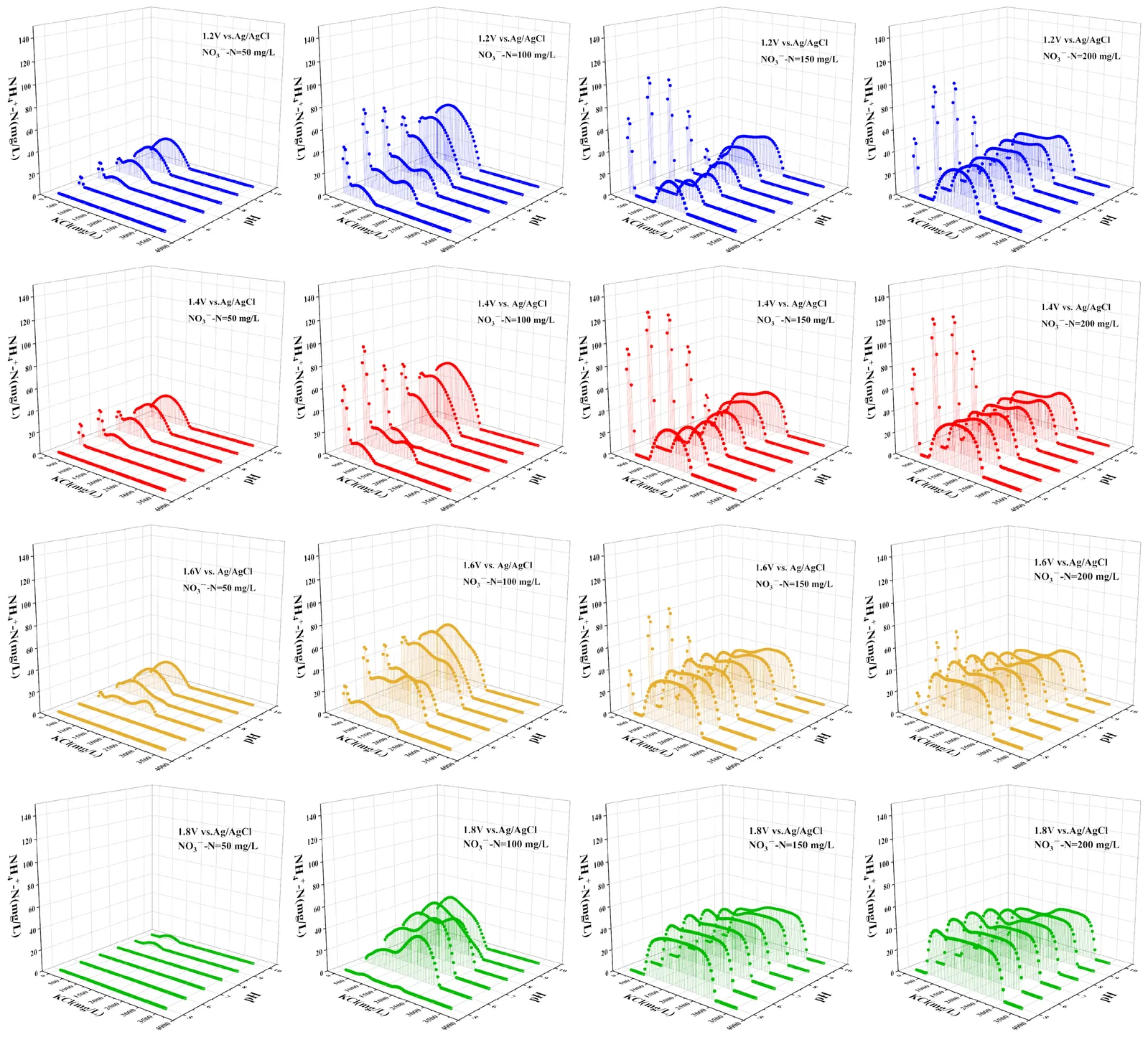
Three-dimensional predictions of NH_4_^+^-N concentration at different voltages, initial NO_3_^−^-N concentrations, KCl concentrations and pH values (160 min).

**Figure 13:**
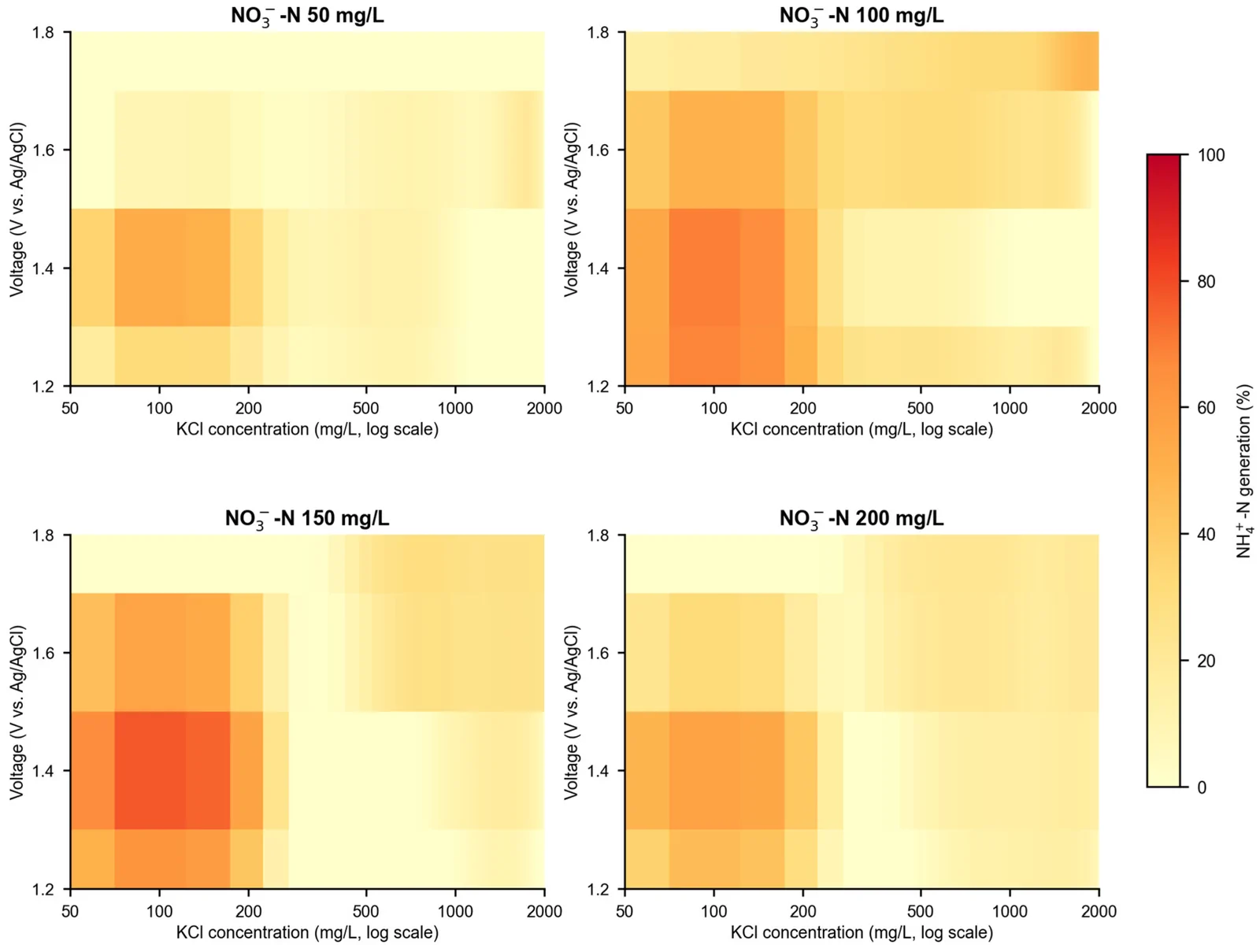
FNN-predicted NH_4_^+^-N yield as a function of initial NO_3_^−^-N concentration (50, 100, 150 and 200 mg/L), KCl concentration and voltage at pH 7.

**Table 1:** Free residual chlorine and combined residual chlorine at different KCl concentrations.

KCl (mg/L)	BiOCl/DSA Anode		DSA Anode	
	Free Residual Chlorine (mg/L)	Combined Residual Chlorine (mg/L)	Free Residual Chlorine (mg/L)	Combined Residual Chlorine (mg/L)
500	1.6	0.7	0.91	0.98
1000	2.8	1.2	0.32	2.31
1500	3	1.4	0.35	2.61
2000	7.4	2.4	1.65	1.31
2500	21.7	0.9	2.66	1.89
3000	10.1	0.2	1.98	2.23

**Table 2:** Performance metrics of different models on the training and test sets.

Model	Training Set				Test Set			
	MAE	MSE	RMSE	R^2^	MAE	MSE	RMSE	R^2^
BP	0.8717	1.5097	1.1625	0.8161	0.7831	1.2077	1.0791	0.8207
GA-BP	0.7388	1.1141	1.0148	0.8456	0.7493	0.9528	0.9714	0.8500
PSO-BP	0.7832	1.0345	0.9974	0.8407	0.7431	1.0431	1.0031	0.8307
FNN	0.4852	0.3483	0.5902	0.9564	0.3560	0.3186	0.5645	0.9507

Note: All metrics represent averages obtained from 30 random 80:20 training test splits. MAE, MSE, and RMSE were calculated using the scale-transformed output data.

## Data Availability

The data presented in this study are available in the article and Supplementary Materials.

## Supplementary Materials

The following supporting information can be downloaded at https://www.mdpi.com/article/10.3390/nano16191219/s1, Section S1: Preparation of the ZVI/Co_3_O_4_/GF electrode; Section S2: Characterization methods; Section S3: Electrochemical measurements; Section S4: Electrochemical experiments; Section S5: Architecture of the back-propagation neural network (BP) model; Section S6: Architecture of the genetic-algorithm-optimized back-propagation neural network (GA-BP) model; Section S7: Architecture of the particle-swarm-optimized back-propagation neural network (PSO-BP) model; Section S8: Architecture of the fuzzy neural network (FNN) model; Section S9: Model evaluation metrics; Figure S1: Preparation of the BiOCl/DSA electrode by wet-chemical synthesis; Figure S2: Standard topology of the FNN model; Figure S3: TC removal efficiencies with (a) different anodes, (b) different initial NO_3_^−^-N concentrations, (c) different initial TC concentrations, (d) different initial pH values, (e) different cathode-potentials magnitude, and (f) different KCl concentrations; Figure S4: Effect of TBA, KBrO_3_, and EDTA on TC degradation (C/C_0_) in the electrochemical system; Figure S5: FNN-predicted NH_4_^+^-N concentration distributions as functions of KCl concentration and cathode potential at different initial NO_3_^−^-N concentrations and pH values; Table S1: Data samples for predicting NH_4_^+^-N production during NO_3_^−^-N reduction.
