# Supplementary material for: Machine Learning-Assisted Analysis of Chloride-Enhanced BiOCl/DSA Anode for Synergistic Denitrification and Tetracycline Degradation: Performance, Mechanisms, and Product Regulation
Source: Nanomaterials (Basel). 2026 Sep 27;16(19):1219. doi: 10.3390/nano16191219 (PMC13666688; doi:10.3390/nano16191219)
Supplement: Supplementary file 1 [file nanomaterials-16-01219-s001.zip › nanomaterials-4581311-supplementary.pdf]

Machine learning-assisted analysis of chloride-enhanced BiOCl/DSA anode for synergistic denitrification and tetracycline degradation: Performance, mechanisms, and product regulation

Yuankun Liu*, Gangyi Sun, Zonglin Li, Heyang Wang, Yufeng He

Municipal Engineering Department, College of Civil Engineering and Architecture, Beijing University of Technology, Beijing 100124, P.R China

E-mails for all authors:

Yuankun Liu, liuyuankun@bjut.edu.cn

Gangyi Sun, 15544216679@163.com

Zonglin Li, 13673881428@163.com

Heyang Wang, wangheyang2007@163.com

Yufeng He, 15114927120@163.com

***Corresponding author**

Tel: +86-10-6739-1726; Fax: +86-10-6739-1726;

E-mail: liuyuankun@bjut.edu.cn

Supplementary data

Section S1. Preparation of the ZVI/Co_3O_4/GF electrode

Section S2. Characterization methods

Section S3. Electrochemical measurements

Section S4. Electrochemical experiments

Section S5. Architecture of the back-propagation neural network (BP) model

Section S6. Architecture of the genetic-algorithm-optimized back-propagation neural network (GA-BP) model

Section S7. Architecture of the particle-swarm-optimized back-propagation neural network (PSO-BP) model

Section S8. Architecture of the fuzzy neural network (FNN) model

Section S9. Model evaluation metrics

Fig. S1. Preparation of the BiOCl/DSA electrode by wet-chemical synthesis

Fig. S2. Standard topology of the FNN model.

Fig. S3. TC removal efficiencies with (a) different anodes, (b) different initial NO_3^--N concentrations, (c) different initial TC concentrations, (d) different initial pH values, (e) different cathode-potentials magnitude, and (f) different KCl concentrations.

Fig. S4. Effect of TBA, $KBrO_3$, and EDTA on TC degradation (C/C_0) in the electrochemical system.

Fig. S5. FNN-predicted NH_4^+-N concentration distributions as functions of KCl concentration and cathode potential at different initial NO_3^--N concentrations and pH values.

Table S1. Data samples for predicting NH_4^+-N production during NO_3^--N reduction

Section S1. Preparation of the ZVI/Co_3O_4/GF electrode

Preparation of the Co_3O_4-based electrode: The graphite-felt substrate (3.0 × 4.0 cm^2) was pretreated by ultrasonication in acetone, 1 M HCl, and distilled water for 20 min each. The electrode was then prepared by electrodeposition followed by calcination. An electrolyte containing 0.5 M H_3BO_3, 0.1 M $Co(NO_3)_2 \cdot 6H_2O$, and 2.0 g L^{-1} sodium dodecyl sulfonate ($C_{12}H_{25}SO_3Na$) was used. The pretreated substrate, a Pt plate, and an Ag/AgCl electrode served as the working, counter, and reference electrodes, respectively. Electrodeposition was performed at −2.0 V versus Ag/AgCl. After deposition, the electrode was rinsed with deionized water and dried at 60 °C. It was subsequently calcined at 500 °C for 2 h in a tube furnace at a heating rate of 5 °C min^{-1}, cooled to room temperature, rinsed, and stored for subsequent use.

Preparation of the ZVI/Co_3O_4/GF electrode: An electrodeposition electrolyte was prepared by dissolving 1.23 g boric acid, 3.3 g ammonium sulfate, 0.05 g glucose pentahydrate, 0.05 g ascorbic acid, 0.025 g sodium dodecyl sulfonate, and 1.65 g ferrous sulfate in 50 mL water. After purging the electrolyte with N_2 for 60 min, the calcined Co_3O_4/GF electrode, a Pt plate, and an Ag/AgCl electrode were used as the working, counter, and reference electrodes, respectively. Electrodeposition was conducted at the specified potential while N_2 purging was maintained throughout the deposition. The resulting electrode was washed several times with ethanol to minimize oxidation and vacuum-dried at 60 °C before use.

Section S2. Characterization methods

The surface morphology of the electrode samples was characterized using an Apreo C field-emission scanning electron microscope (SEM; Thermo Fisher Scientific). The deposition, crystal morphology, and surface coverage of BiOCl on the DSA were examined to evaluate changes in the electrode microstructure after modification.

Section S3. Electrochemical measurements

Electrochemical measurements were conducted in a quartz electrochemical cell using a potentiostat and a standard three-electrode configuration. BiOCl/DSA and ZVI/Co_3O_4/GF were used as the anode and cathode, respectively. For the CV and LSV measurements, the exposed geometric areas of the working and counter electrodes were both 1.0 cm^2, and the interelectrode distance was 10 mm. Cyclic voltammetry (CV) and linear sweep voltammetry (LSV) were performed using an electrochemical workstation. Anodic LSV measurements were conducted using the DSA or BiOCl/DSA anode as the working electrode in electrolytes with different KCl concentrations. The potentials were reported versus RHE. CV was conducted from −0.5 to 0 V in a solution containing 0.1 M Na_2SO_4 and 100 mg/L NO_3^--N. All potentials were converted to the reversible hydrogen electrode (RHE) scale.Potentials were converted according to E_{RHE}=$E_{Ag/AgCl}$+0.197+0.0592pH at 25 °C.

Section S4. Electrochemical experiments

Electrochemical reduction experiments were conducted in a single-chamber quartz cell using a standard three-electrode configuration. BiOCl/DSA and ZVI/Co_3O_4/GF were used as the anode and cathode, respectively. In the 100 mL batch reactor, both the BiOCl/DSA anode and the ZVI/Co_3O_4/GF cathode had physical dimensions of 3.0 cm × 4.0 cm, corresponding to a projected geometric area of 12.0 cm^2 per face. Nitrate reduction was performed at a cathode potential of −1.61 V versus Ag/AgCl in 100 mL solution containing 100 mg L^{-1} NO_3^--N and 0.5 M Na_2SO_4. The initial pH was 7.0, and the reaction time was 160 min, with samples collected every 20 min. NO_3^--N, NO_2^--N, and NH_4^+-N concentrations were measured using a UV-1900 UV–visible spectrophotometer. The analytical procedures and equations used to calculate nitrogen-species removal, product yields, and tetracycline (TC) removal are described below.

(1) NO_3^--N was determined using the dual-wavelength method. A 1-mL sample was diluted to 50 mL with deionized water, after which 1 mL of 1 mol/L HCl and 0.1 mL of sulfamic acid solution were added sequentially. After thorough mixing, absorbance was measured at 220 nm (A_1) and 270 nm (A_2), and the concentration was calculated from the calibration curve.

(2) NO_2^--N was determined by the sulfanilamide–N-(1-naphthyl)ethylenediamine spectrophotometric method. A 1-mL sample was diluted to 50 mL with deionized water, mixed with 1 mL of colour reagent, and allowed to develop for 20 min. Absorbance was then measured at 540 nm, and the concentration was calculated from the calibration curve.

The nitrite colour reagent was prepared by adding 250 mL deionized water and 50 mL phosphoric acid solution to a 500-mL beaker, followed by 20 g sulfanilamide. After complete dissolution, 1 g N-(1-naphthyl)ethylenediamine dihydrochloride was added and dissolved. The solution was diluted to 500 mL with water, mixed thoroughly, stored in an amber bottle, and refrigerated at 4 °C until use.

(3) NH_4^+-N was determined using the Nessler-reagent spectrophotometric method. A 1-mL sample was diluted to 50 mL, and 1 mL of potassium sodium tartrate solution

(0.5 g/mL) and 1 mL of Nessler reagent were added. After mixing and colour development for 10 min, absorbance was measured at 420 nm and the concentration was calculated from the calibration curve.

(4) Calculation of removal efficiencies and product yields: DIN removal was calculated from the initial NO_3^--N concentration and the measured residual NO_3^--N, NO_2^--N, and NH_4^+-N concentrations. The unaccounted fraction was not assigned to a specific nitrogen product.[1].

$$NO_3^-\text{-N removal efficiency } R(NO_3^-\text{-N})=\frac{C(NO_3^-\text{-N})_0-C(NO_3^-\text{-N})_t}{C(NO_3^-\text{-N})_0}\times100\% \quad (1)$$

$$NH_4^+\text{-N selectivity/yield } FE(NN_4^+\text{-N})=\frac{C(NH_4^+\text{-N})_t}{C(NO_3^-\text{-N})_0-C(NO_3^-\text{-N})_t}\times100\% \quad (2)$$

$$\text{DIN removal efficiency } R(DIN\text{-N})=\frac{C(NO_3^-\text{-N})_0-C(NO_3^-\text{-N})_t-C(NH_4^+\text{-N})_t-C(NO_2^-\text{-N})_t}{C(NO_3^-\text{-N})_0}\times100\% \quad (3)$$

where $C(NO_3^-\text{-N})_0$ is the initial NO_3^--N concentration, and $C(NO_3^-\text{-N})_t$, $C(NH_4^+\text{-N})_t$, and $C(NO_2^-\text{-N})_t$ are the corresponding concentrations at time t.

(5) TC concentration was determined by UV–visible spectrophotometry at 365 nm, using deionized water as the blank. Concentrations were obtained from the corresponding calibration curve.

Section S5. Architecture of the back-propagation neural network (BP) model

The BP neural network used reaction time, KCl concentration, initial NO_3^--N concentration, cathode-potential magnitude, and pH as input variables and NH_4^+-N concentration as the output variable. The input and output layers contained five nodes and one node, respectively. Before modelling, the input and output data were normalized as follows:

$$x' = \frac{x - x_{min}}{x_{max} - x_{min}} \tag{5}$$

The hidden-layer output was calculated as:

$$h_j = f\left(\sum_{i=1}^{5} w_{ij} x_i + b_j\right) \tag{6}$$

The output-layer prediction was calculated as:

$$y = g\left(\sum_{j=1}^{m} v_j h_j + b_0\right) \tag{7}$$

where x_i is an input variable; h_j is the output of hidden-layer node j; w_{ij} and v_j are the connection weights from the input layer to the hidden layer and from the hidden layer to the output layer, respectively; b_j and b_0 are bias terms; and f and g are the activation functions of the hidden and output layers, respectively. The weights and biases were updated through error back-propagation until the specified error threshold or maximum number of iterations was reached.

Section S6. Architecture of the genetic-algorithm-optimized back-propagation neural network (GA-BP) model

The GA-BP model used a genetic algorithm to optimize the initial weights and biases of the BP neural network. All weights and biases to be optimized were first encoded into an individual chromosome:

$$X = [w_1, w_2, ..., w_m, b_1, b_2, ..., b_k] \qquad (8)$$

The fitness function was constructed from the BP-network prediction error:

$$\text{Fitness} = \frac{1}{(\text{MSE} + \varepsilon)} \qquad (9)$$

where ε is a small constant used to prevent division by zero. The genetic algorithm searched for the optimal combination of weights and biases through selection, crossover, and mutation. When the maximum number of generations was reached or the fitness converged, the optimal individual was used to initialize the BP neural network, which was then further trained using the BP algorithm to obtain the GA-BP prediction model.

Section S7. Architecture of the particle-swarm-optimized back-propagation neural network (PSO-BP) model

The PSO-BP model used particle-swarm optimization to optimize the initial weights and biases of the BP neural network. Each particle represented a set of BP-network parameters:

$$X_i = [w_1, w_2, ..., w_m, b_1, b_2, ..., b_k] \tag{10}$$

Particle velocity and position were updated as follows:

$$v_i(t+1) = \omega v_i(t) + c_1 r_1 [p_{best\_i} - x_i(t)] + c_2 r_2 [g_{best} - x_i(t)] \tag{11}$$

$$x_i(t+1) = x_i(t) + v_i(t+1) \tag{12}$$

where ω is the inertia weight; c_1 and c_2 are learning factors; r_1 and r_2 are random numbers between 0 and 1; $pbest_i$ is the personal best position of particle i; and gbest is the global best position. After iteration, the weights and biases corresponding to the global best particle were used to initialize the BP network, which was then further trained using the BP algorithm to obtain the PSO-BP prediction model.

Section S8. Architecture of the fuzzy neural network (FNN) model

The fuzzy neural network (FNN) combines fuzzy logic (FL) with a neural network (NN) and uses gradient descent for iterative parameter updating. The standard FNN architecture comprises five layers: an input layer, a membership-function layer, a rule layer, a defuzzification layer, and an output layer.

Input layer: This layer contains n neurons and introduces the n-dimensional input vector X into the model.

$$X = [x_1, x_2, \ldots, x_n] \quad (13)$$

Membership-function layer: This layer fuzzifies the input signals. Different nodes represent different fuzzy membership functions. Gaussian functions were used as the membership functions in this study.

$$u_{ij} = \exp\left[-\frac{(x_i - c_{ij})^2}{\sigma_{ij}^2}\right] i = 1,2\ldots, n j = 1,2\ldots, m \quad (14)$$

where c_{ij} and σ_{ij} denote the centre and width of the Gaussian membership function, respectively, and m is the number of membership functions.

Fuzzy-rule layer: Each neuron in this layer represents one fuzzy rule, and the number of neurons corresponds to the number of fuzzy rules. Rule activation was calculated as follows:

$$f_j(t) = \prod_{i=1}^{n} u_{ij}(t) \quad (15)$$

Defuzzification layer: This layer combines the rule activations and converts the fuzzy quantities into crisp values.

$$\bar{f}_j(t) = \frac{f_j(t)}{\sum_{j=1}^{m} f_j(t)} \quad (16)$$

Output layer: This layer calculates a weighted sum of the defuzzified outputs.

$$y(t) = \sum_{j=1}^{m} w_j \times \bar{f}(t) \quad (17)$$

where w_j is the connection weight.

Section S9. Model evaluation metrics

All models used the same dataset, input/output variables, and training/test split. Model performance was evaluated using MAE, MSE, RMSE, and R^2, calculated as follows:

$$\mathrm{MAE}\left(y_i,\hat{y}i\right)=\frac{1}{n}\sum_{i=1}^{n}|y_i-\hat{y}i| \quad (18)$$

$$\mathrm{MSE}\left(y_i,\hat{y}i\right)=\frac{1}{n}\sum_{i=1}^{n}(y_i-\hat{y}i)^2 \quad (19)$$

$$\mathrm{RMSE}\left(y_i,\hat{y}i\right)=\sqrt{\frac{1}{n}\sum_{i=1}^{n}(y_i-\hat{y}i)^2} \quad (20)$$

$$R^2=1-\frac{\sum_{i=1}^{n}(y_i-\hat{y}i)^2}{\sum_{i=1}^{n}(y_i-\bar{y})^2} \quad (21)$$

where y_i is the observed value, $\hat{y}_i$ is the predicted value, and $\bar{y}$ is the mean of the observed values.

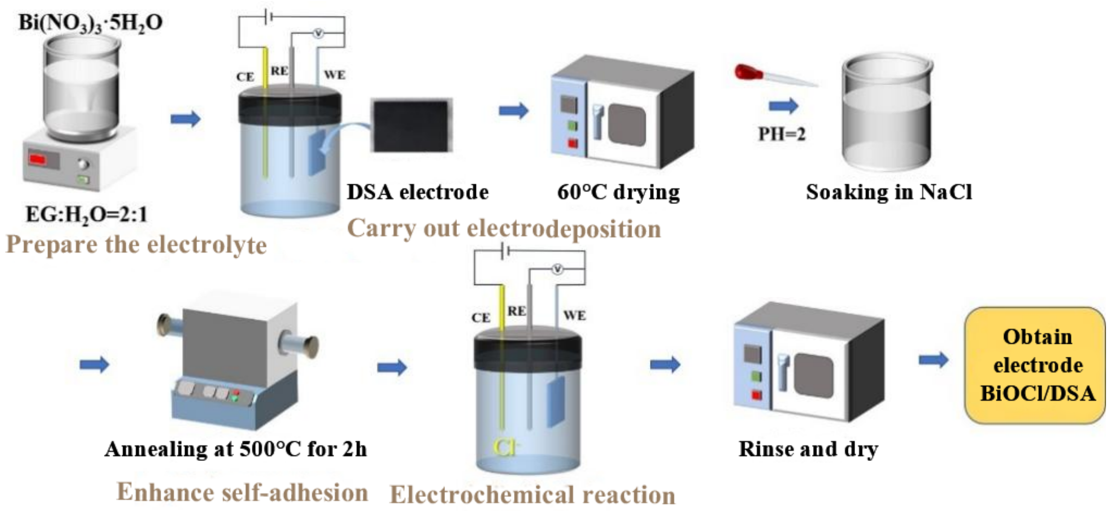

Fig. S1. Preparation of the BiOCl/DSA electrode by wet-chemical synthesis.

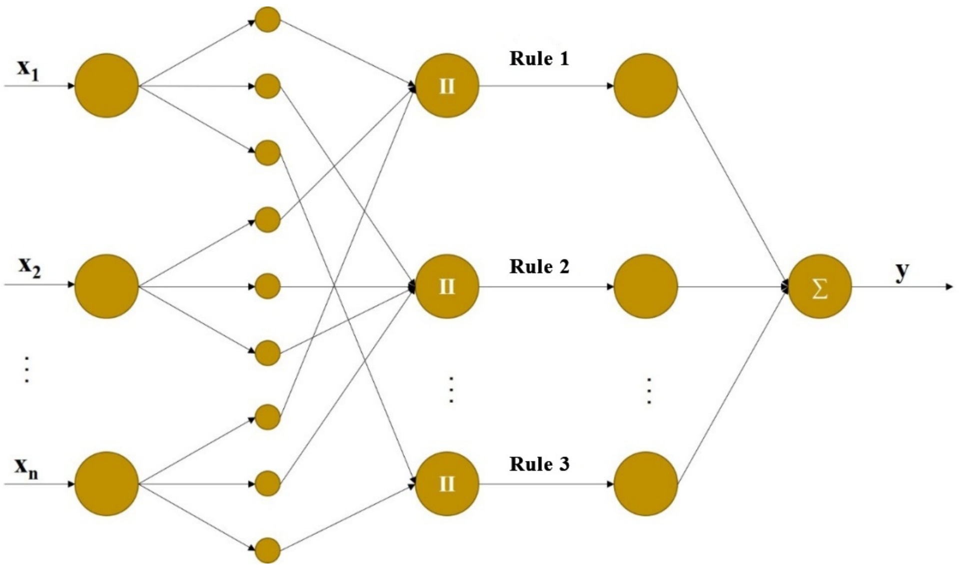

Fig. S2. Standard topology of the FNN model.

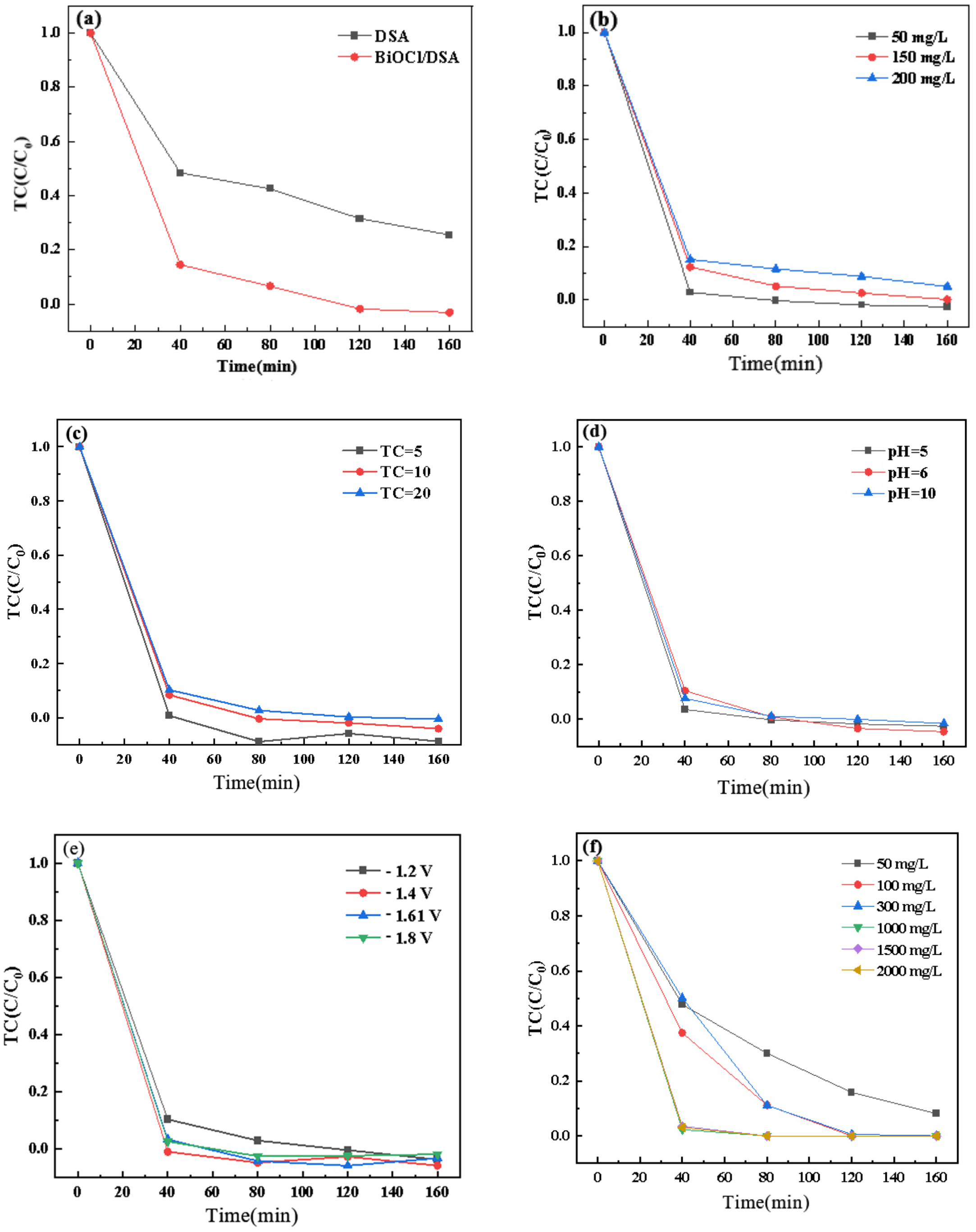

Fig. S3. TC removal efficiencies with (a) different anodes, (b) different initial NO_3^--N concentrations, (c) different initial TC concentrations, (d) different initial pH values, (e) different cathode potentials, and (f) different KCl concentrations.

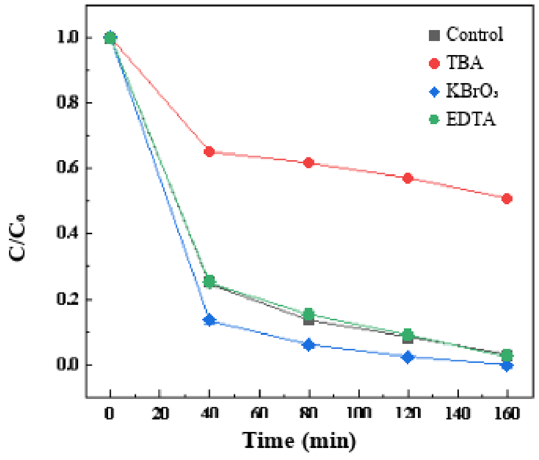

Fig. S4. Effect of TBA, $KBrO_3$, and EDTA on TC degradation (C/C_0) in the electrochemical system.

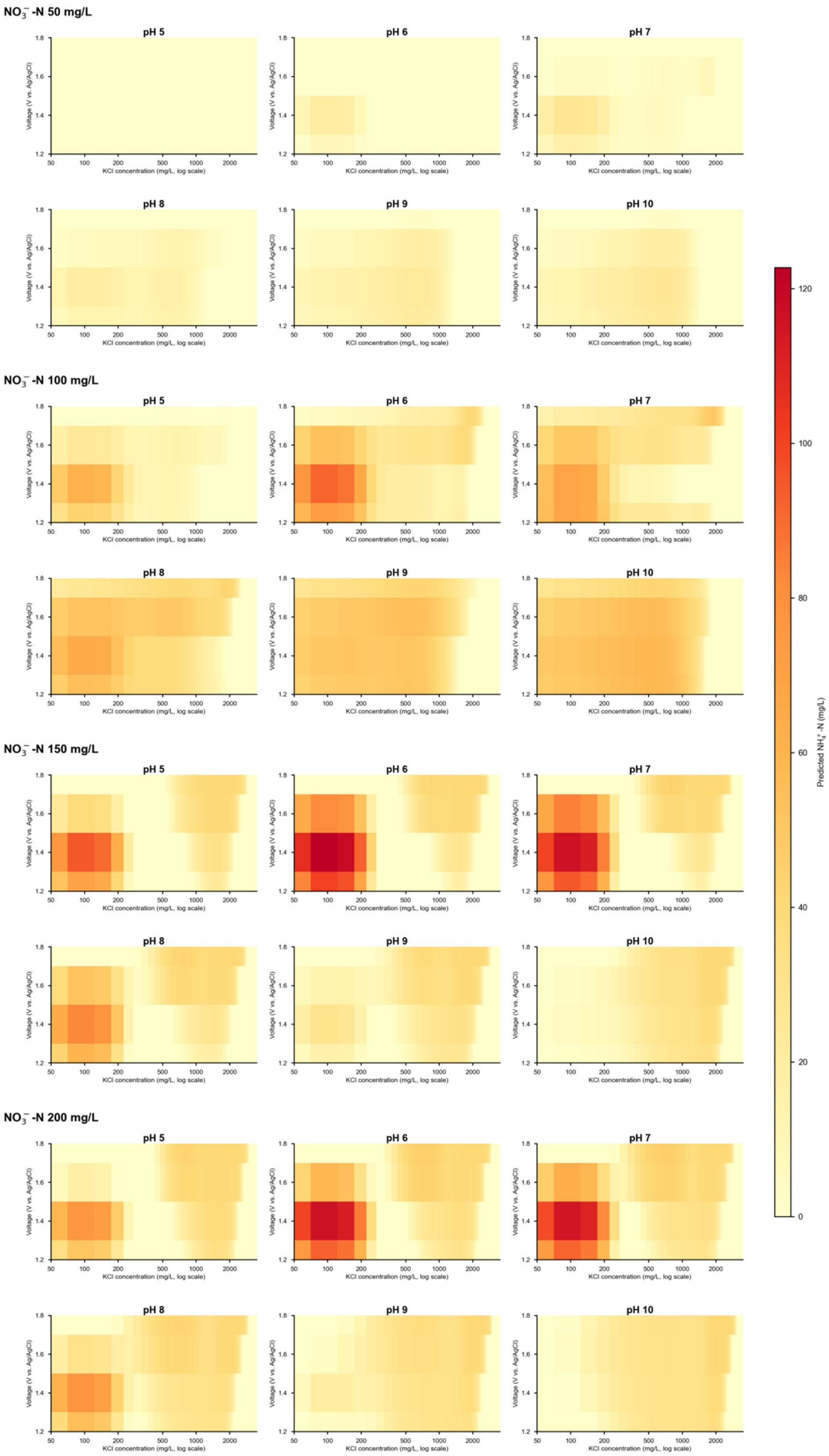

Fig. S5. FNN-predicted NH_4^+-N concentration distributions as functions of KCl concentration and cathode potential at different initial NO_3^--N concentrations and pH values.

Table S1. Data samples for predicting NH_4^+-N production during NO_3^--N reduction

No.	Time（min）	KCl concentration（mg/L）	Initial NO_3^--N concentration（mg/L）	$\|E_c\|$ (V)	pH	NH_4^+-N concentration（mg/L）
1	20	0	100	1.4	7	6.14925
2	40	0	100	1.4	7	35.97015
3	60	0	100	1.4	7	45.37313
4	80	0	100	1.4	7	51.31343
5	100	0	100	1.4	7	44.41791
6	120	0	100	1.4	7	37.8209
7	20	250	100	1.4	7	6.74627
8	40	250	100	1.4	7	14.80597
9	60	250	100	1.4	7	21.37313
10	80	250	100	1.4	7	27.91045
11	100	250	100	1.4	7	33.73134
12	120	250	100	1.4	7	37.73134
13	20	10	100	1.4	7	20.41791
14	40	10	100	1.4	7	40.59701
15	60	10	100	1.4	7	49.79104
16	80	10	100	1.4	7	49.04478
17	100	10	100	1.4	7	47.85075
18	120	10	100	1.4	7	40.47761
19	20	1000	100	1.4	7	0
20	40	1000	100	1.4	7	3.46269
21	60	1000	100	1.4	7	6.23881
22	80	1000	100	1.4	7	9.13433
23	100	1000	100	1.4	7	0
24	120	1000	100	1.4	7	0
25	20	1500	100	1.4	7	16.92537
26	40	1500	100	1.4	7	24.98507
27	60	1500	100	1.4	7	27.28358
28	80	1500	100	1.4	7	27.55224
29	100	1500	100	1.4	7	15.8209
30	120	1500	100	1.4	7	0
31	40	500	100	1.4	7	22.86567
32	80	500	100	1.4	7	53.01493
33	120	500	100	1.4	7	35.79104
34	160	500	100	1.4	7	23.79104
35	40	0	50	1.4	7	11.79104
36	80	0	50	1.4	7	13.67164
37	120	0	50	1.4	7	9.07463
38	160	0	50	1.4	7	0.1194
39	40	250	50	1.4	7	4.2345
40	80	250	50	1.4	7	9.2356

Table S1. Data samples for predicting NH_4^+-N production during NO_3^--N reduction (continued list)

No.	Time（min）	KCl concentration（mg/L）	Initial NO_3^--N concentration（mg/L）	$\|E_c\|$ (V)	pH	NH_4^+-N concentration（mg/L）
41	120	250	50	1.4	7	5.5236
42	160	250	50	1.4	7	0
43	40	10	50	1.4	7	9.79104
44	80	10	50	1.4	7	11.67164
45	120	10	50	1.4	7	7.07463
46	160	10	50	1.4	7	0.094
47	40	1000	50	1.4	7	2.79104
48	80	1000	50	1.4	7	3.95441
49	120	1000	50	1.4	7	0.2347
50	160	1000	50	1.4	7	0.00155556
51	40	1500	50	1.4	7	1.5674
52	80	1500	50	1.4	7	2.354
53	120	1500	50	1.4	7	0.1245
54	160	1500	50	1.4	7	0
55	40	0	200	1.4	7	30.71642
56	80	0	200	1.4	7	47.91045
57	120	0	200	1.4	7	58.47761
58	160	0	200	1.4	7	30.71642
59	40	0	150	1.4	7	20.68657
60	80	0	150	1.4	7	41.13433
61	120	0	150	1.4	7	55.8209
62	160	0	150	1.4	7	47.55224
63	40	0	100	1.4	5	27.37313
64	80	0	100	1.4	5	33.28358
65	120	0	100	1.4	5	27.97015
66	160	0	100	1.4	5	10.14925
67	40	0	100	1.4	6	31.43284
68	80	0	100	1.4	6	55.64179
69	120	0	100	1.4	6	48.83582
70	160	0	100	1.4	6	34.86567
71	40	0	100	1.4	10	55.01493
72	80	0	100	1.4	10	57.73134
73	120	0	100	1.4	10	46.62687
74	160	0	100	1.4	10	36.89552
75	40	0	100	1.2	7	21.34328
76	80	0	100	1.2	7	41.70149
77	120	0	100	1.2	7	31.8806
78	160	0	100	1.2	7	27.79104
79	40	0	100	1.4	7	20.98507

Table S1. Data samples for predicting NH_4^+-N production during NO_3^--N reduction (continued list)

No.	Time（min）	KCl concentration（mg/L）	Initial NO_3^--N concentration（mg/L）	$\|E_c\|$ (V)	pH	NH_4^+-N concentration（mg/L）
80	80	0	100	1.4	7	18.59701
81	120	0	100	1.4	7	5.40299
82	160	0	100	1.4	7	0
83	40	0	100	1.6	7	44.98507
84	80	0	100	1.6	7	45.04478
85	120	0	100	1.6	7	27.10448
86	160	0	100	1.6	7	8.20896
87	40	0	100	1.8	7	41.22388
88	80	0	100	1.8	7	31.46269
89	120	0	100	1.8	7	8.32836
90	160	0	100	1.8	7	8.38806

References

[1] Z. Shen, D. Liu, G. Peng, Y. Ma, J. Li, J. Shi, J. Peng, L. Ding, Electrocatalytic reduction of nitrate in water using Cu/Pd modified Ni foam cathode: High nitrate removal efficiency and N2-selectivity, Separation and Purification Technology 241 (2020). https://doi.org/10.1016/j.seppur.2020.116743.
